# Behaviourally driven closed-loop beta-tACS enhances beta activity and motor behaviour

**DOI:** 10.1038/s41467-026-75799-8

**Published:** 2026-07-21

**Authors:** Min Wu, Zeyu Xu, Melanie K. Fleming, Nicholas Shackle, Lara Biller, Faye Tabone, Pei-Ling Wong, Caroline Nettekoven, Andrew Sharott, Catharina Zich, Charlotte J. Stagg

**Affiliations:** 1 Oxford Centre for Integrative Neuroimaging, FMRIB, Nuffield Department of Clinical Neurosciences, University of Oxford, Oxford, UK; 2 Medical Research Council Brain Network Dynamics Unit, Nuffield Department of Clinical Neuroscience, University of Oxford, Oxford, UK; 3 Department of Physical Therapy and Assistive Technology, National Yang Ming Chiao Tung University, Taipei, Taiwan, ROC; 4 Department of Experimental Psychology, University of Oxford, Oxford, UK

**Keywords:** Motor cortex, Neurophysiology

## Abstract

Movement-related beta event-related synchronization (ERS) has been linked to motor control and learning, showing potential as a therapeutic target for those with movement deficits, such as stroke survivors. However, whether directly modulating beta ERS can causally influence motor performance remains unclear, largely due to the lack of methods designed to specifically target this neural activity. To address this gap, we developed a behaviourally-driven, closed-loop transcranial alternating current stimulation (tACS) approach to target movement-related beta ERS during a visuomotor adaptation task. We found that the behaviourally-driven, closed-loop beta-tACS specifically enhances beta ERS without affecting beta event-related desynchronization (ERD). Critically, this targeted enhancement significantly improves retention of motor adaptation. These findings support a functional role of beta ERS in motor behaviour and suggest that behaviourally-driven beta-tACS may provide a promising approach to further test the mechanistic links between beta ERS and behavioural outcomes in clinical populations.

## Abstract (web summary)

Wu and colleagues developed movement-triggered brain stimulation that increased beta-band brain activity and improved retention of a newly learned motor adaptation.

## Introduction

From performing keyhole surgery to playing Tchaikovsky’s Piano Concertos, humans are uniquely able to learn and perform skilled movements. Even in our daily lives, being able to type, dress and feed ourselves requires precise timing of neural activity to learn and refine motor patterns. The extent of motor impairment in patients with neurological disorders has been linked to the degree of disruption in this neural activity, making it a highly promising target for therapeutic rehabilitation. Here, we present the evidence that targeting the neural activity underpinning motor patterns using closed-loop non-invasive brain stimulation can modulate that neural activity, leading to behavioural improvements and offering a promising approach for therapeutic interventions in clinical populations.

Human movement is dependent on changes in neural activity within the cortico-basal ganglia circuits in the beta frequency range (13–30 Hz). Specifically, movements are characterised by a decrease in movement-related beta activity during movement execution (event-related desynchronization, ERD) and an increase following movement termination (event-related synchronization, ERS)^1,2^. The amplitude of movement-related beta activity patterns relates to movement kinematics and motor learning in animals and in healthy adults^3–6^, and alterations of movement-related beta activity are observed in a range of neurological conditions. For example, the reduction of the beta ERD mediates the disordered movement characteristic of Parkinson’s disease, and treatments that alleviate motor symptoms appear to do so via restoring movement-related beta activity^7–9^. Similarly, the beta ERS is decreased after stroke and increases during motor recovery, with people who recover motor function having a stronger beta ERS than those who do not recover motor function^10–12^. Indeed, non-invasive brain stimulation approaches aiming at improving motor recovery after stroke also increase the beta ERS^13,14^. However, the causality of these relationships has been impossible to determine: does improved movement lead to increased ERS, or does increased ERS lead to improved movement?

**Fig. 1:**
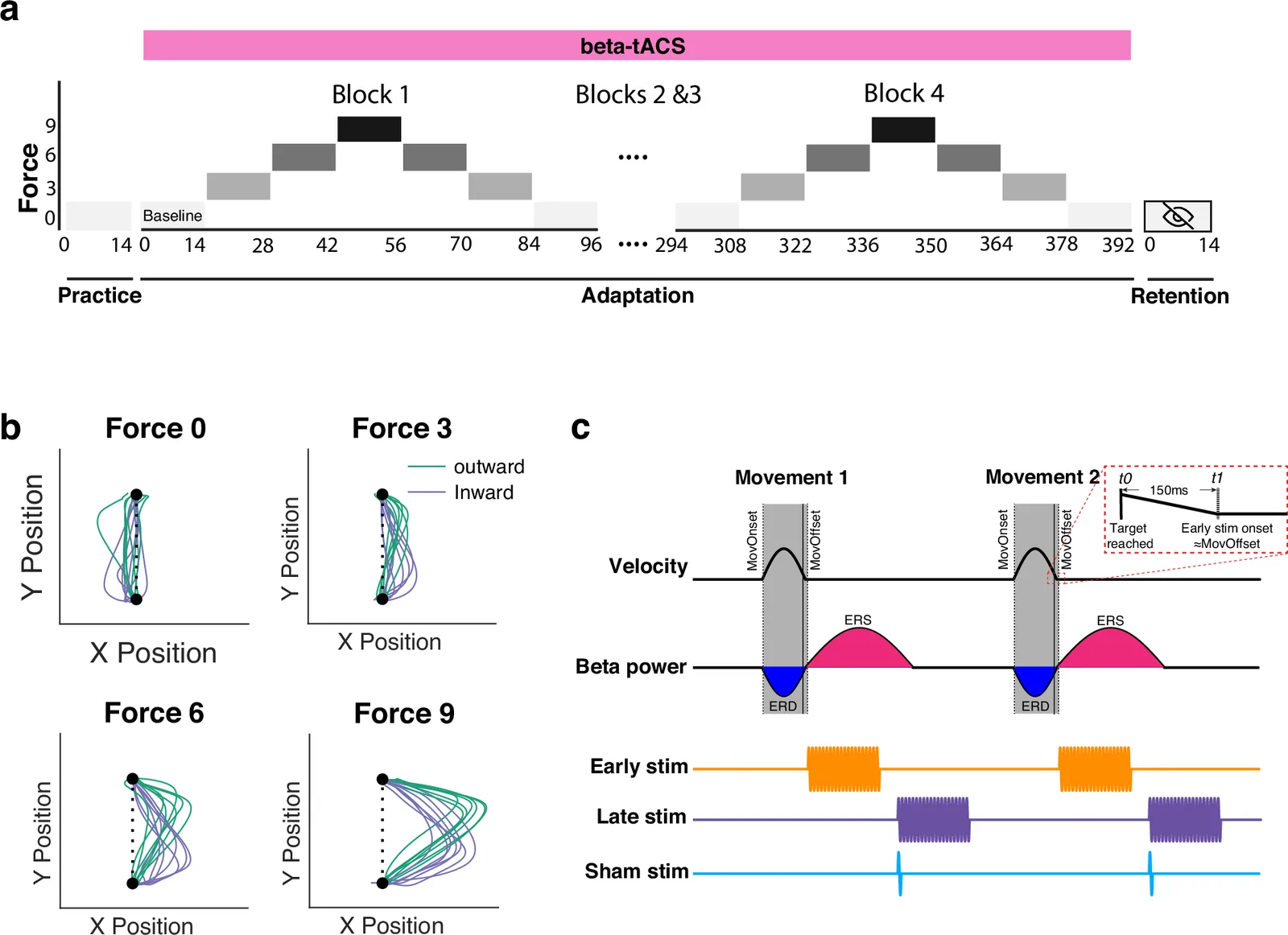
Experimental setup and stimulation protocol. **a** Schematic of the target-reaching motor task. The task consisted of three phases: a practice phase, an adaptation phase with a lateral force field perturbation, and a retention phase with no force field and without visual feedback. **b** Example movement trajectories from a single participant at different force field magnitudes (0, 3, 6 and 9). Green trajectories depict outward/upward movements and purple trajectories depict inward/downward movements. **c** Overview of the behaviourally driven closed-loop tACS. In the early stimulation condition (orange), 20 Hz tACS was triggered following movement offset (i.e. *t*_1_, 150 ms after target reached [*t*_0_]) and lasted 2 s. In the late stimulation condition (purple), tACS was triggered 2.5 s after movement offset and lasted 2 s. In the sham condition (blue), stimulation was triggered 2.5 s after movement offset but lasted only 100 ms. Time *t*_0_ was defined as the moment that the cursor was moved into the target area, and time *t*_1_ was defined as the moment that the cursor remained continuously within the target area for 150 ms.

We address this question here by developing a behaviourally-driven closed-loop non-invasive brain stimulation approach that triggers transcranial alternating current stimulation (tACS) in real time to target a specific brain state. tACS applies sinusoidally varying electrical current through the scalp, and can modulate endogenous neural activity at specific frequencies^15^. Traditionally, tACS involves applying continuous stimulation irrespective of the ongoing brain state^16–18^. However, recent studies indicate that the effectiveness of brain stimulation depends on the underlying brain state at the moment of stimulation^19,20^, among other factors such as participant anatomy^21^. This has driven the development of closed-loop, state-dependent stimulation that adjusts stimulation in real time. We have developed a behaviourally-driven closed-loop beta-tACS approach, where stimulation was triggered by the participant’s behaviour. Behaviourally-driven closed-loop brain stimulation may offer advantages over neurally-driven approaches by targeting behaviourally-related neural activity more directly, which can potentially lead to more effective outcomes. In our case, we use the end of the movement, in line with the beta ERS (Fig. 1), to trigger beta-tACS, aiming at enhancing the beta ERS and thereby improve motor control.

Here, 24 young healthy adults completed three separate sessions in a double-blind, sham-controlled crossover design involving three stimulation conditions: (i) an early stimulation condition, delivering 2 s of beta-tACS at movement offset; (ii) a late stimulation condition, delivering the same stimulation 2.5 s after movement offset; and (iii) a sham stimulation condition, delivering brief (100 ms stimulation) also at 2.5 s after movement offset. To investigate the stimulation effects on both behavioural and neural levels, we used a target-reaching visuomotor adaptation task with a Kinarm robotic system to assess motor behaviour, and simultaneously recorded electroencephalography (EEG) to measure beta activity in stimulation-free periods. We hypothesised that behaviourally-triggered, closed-loop beta tACS delivered at movement offset (i.e. the early stimulation condition) would enhance beta ERS in the sensorimotor cortex and facilitate motor performance in a visuomotor adaptation task.

## Results

### Behaviourally triggered beta tACS enhances post-movement beta ERS but not ERD

**Fig. 2:**
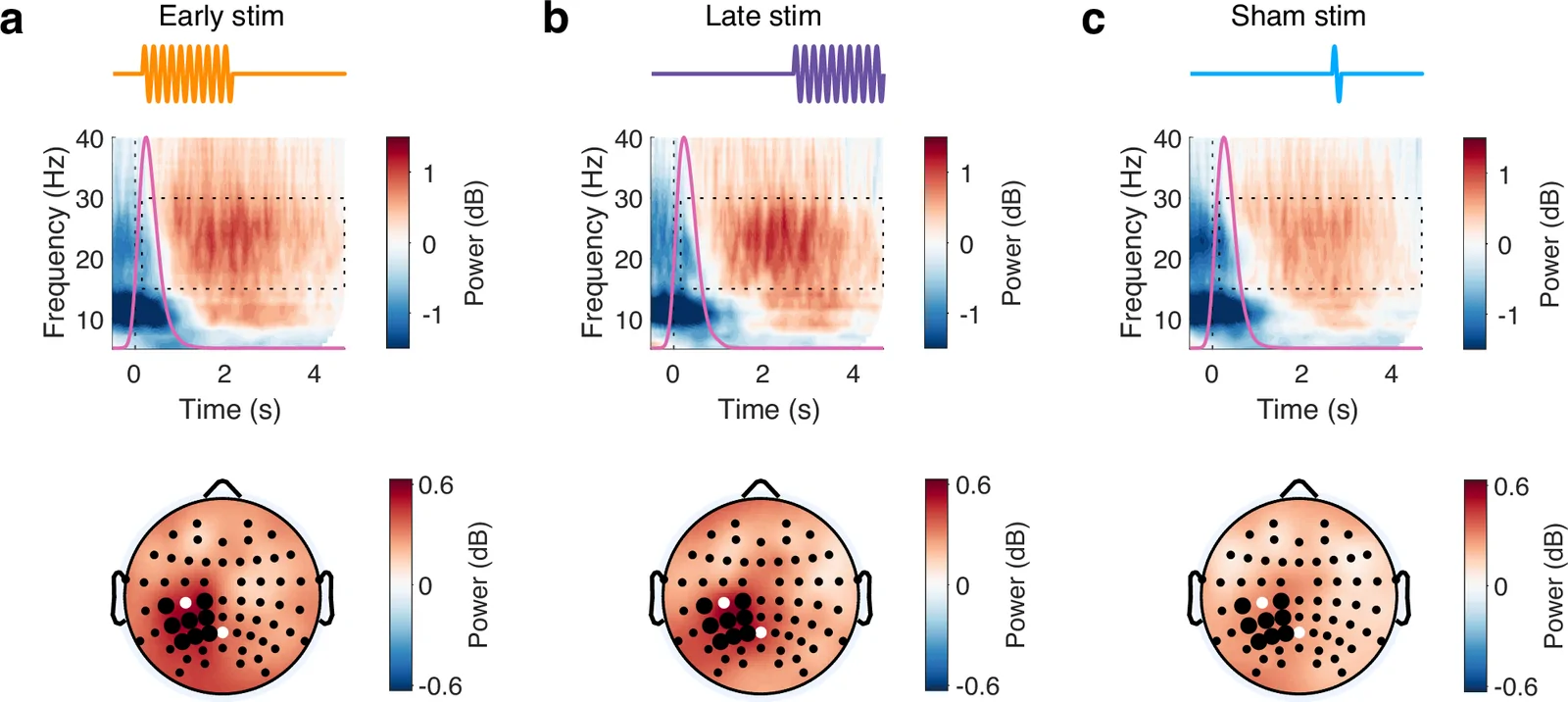
Post-movement time–frequency responses across stimulation conditions. **a** tACS and time-frequency response aligned to target reached (*t*_0_ = 0 s) for the early stimulation condition (*n* = 24 participants). The power was averaged across sensors covering the contralateral sensorimotor cortex (highlighted in bold in the bottom panel). The magenta curve shows the distribution of the movement offsets across participants. The black dashed rectangle indicates the time–frequency window used to quantify post-movement beta ERS. The scalp topography shows a contralateral distribution of beta ERS. Black dots mark electrodes in the predefined sensorimotor area; white dots indicate stimulation electrodes (C3 and Pz). **b** Similar as (**a**), for the late stimulation condition (*n* = 24 participants). **c** Similar as (**a**), for the sham stimulation condition (*n* = 24 participants). All EEG analyses were performed on non-stimulated trials, and trials containing stimulation artefacts were rejected.

We first wanted to investigate the effects of behaviourally-triggered beta tACS on the post-movement beta ERS. EEG was recorded during the performance of the visuomotor adaptation task and was analysed from those trials where tACS was not applied; all subsequent EEG results are based on non-stimulated epochs. As expected, after participants reached the target, there was a robust increase in beta-band power (beta ERS; 15–30 Hz) over the left sensorimotor area, contralateral to the upper limb performing the movement (Fig. 2).

**Fig. 3:**
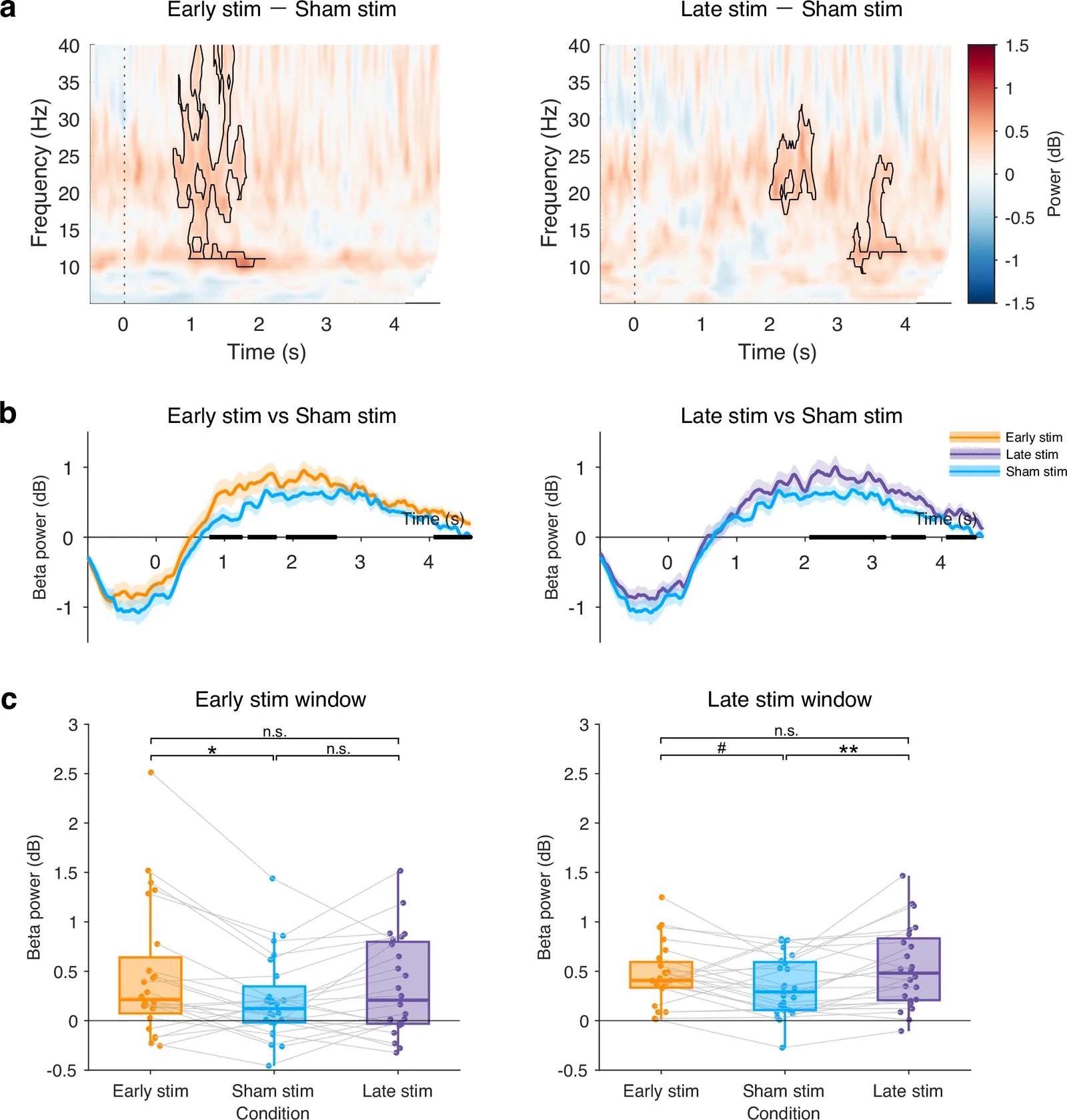
Modulation of post-movement beta activity by stimulation conditions. **a** Time-frequency response difference in the sensorimotor cortex between conditions (*n* = 24 participants). The black outlines indicate significant clusters with *p* < 0.05 by cluster-based permutation test (one-sided, 1000 permutations). **b** Time course of beta power (15–30 Hz) averaged over the contralateral sensorimotor area, aligned to target reached (*t*_0_ = 0 s). Left: comparison between early (orange) and sham stimulation (blue). Right: comparison between late (purple) and sham (blue) stimulation. Shaded areas indicate s.e.m. Black horizontal bars indicate time intervals with significant differences between conditions, determined by cluster-based permutation testing (*p* < 0.05). **c** Mean beta power extracted from the early (left) and late (right) stimulation windows (*n* = 24 participants per condition; repeated measures). Each dot represents an individual participant; grey lines link participant data across conditions. The horizontal line within each box indicates the median; the bottom and top edges of the box indicate the first and third quartiles, respectively; and the whiskers indicate the most extreme values within 1.5 times the IQR. Compared to sham stimulation, beta ERS was significantly enhanced following early stimulation in the early window (*t* = 2.975, *p* = 0.013, FDR-corrected) and following late stimulation in the late window (*t* = 3.296, *p* = 0.005, FDR-corrected). n.s. non-significant, ^#^*p* < 0.1, *p < 0.05 and **p < 0.01. dB decibel, IQR interquartile range.

To determine whether stimulation modulated motor-related beta activity, we performed cluster-based permutation testing on the time-frequency responses in the contralateral sensorimotor cortex (Fig. 3a), and on the mean beta power in the contralateral sensorimotor cortex (Fig. 3b). These analyses demonstrated that beta activity in the early stimulation session differed significantly from beta activity in the sham session primarily within the early post-movement window, whereas beta activity in the late stimulation session differed significantly from the sham session in the late window.

These findings were further supported when we calculated mean beta power within two stimulation-aligned time windows: the early stimulation window (0–2 s relative to *t*_1_ [~movement offset]) and a late stimulation window (2.5–4.5 s relative to *t*_1_), and compared across stimulation conditions. In the early stimulation window, beta ERS differed significantly across stimulation conditions (*χ*^2^(2) = 9.253, *p* = 0.010; Fig. 3c, left). Beta ERS was significantly higher in the early stimulation session compared to sham (*t*(50.1) = 2.975, *p* = 0.013, FDR-corrected), with no significant differences observed between late stimulation and sham (*t*(50.1) = 1.598, *p* = 0.175, FDR-corrected), or between early and late stimulation (*t*(50.1) = 1.377, *p* = 0.175, FDR-corrected).

In the late stimulation window, there was also a main effect of stimulation condition (*χ*^2^(2) = 11.582, *p* = 0.003; Fig. 3c, right). Beta ERS was significantly enhanced in the late stimulation session compared to sham (*t*(50.1) = 3.296, *p* = 0.005, FDR-corrected), though no significant difference was observed between the early and late stimulation sessions (*t*(50.1) = −1.229, *p* = 0.225, FDR-corrected).

To determine whether these stimulation-related changes were specific to the beta ERS, we investigated the amplitude of the beta ERD in a similar way. As would be expected, there was a significant decrease in beta-band power (i.e. beta ERD) during movement, distributed bilaterally over the sensorimotor area (Supplementary Fig. S1). However, when the mean amplitude of the beta power within the movement period (0–1.5 s relative to movement onset) was calculated and compared across stimulation conditions, no significant differences in beta ERD were observed (*χ*^2^(2) = 2.615, *p* = 0.271; Supplementary Fig. S2). This null effect was further supported by the Bayes Factor (BF_01_ = 20.160), indicating strong evidence for the absence of stimulation effects on beta ERD. Crucially, a comparison of beta activity in the pre-movement baseline window revealed no significant differences across stimulation conditions (*χ*^2^(2) = 1.427, *p* = 0.49; BF_01_ = 35.645; Supplementary Fig. S2d).

Taken together, these results indicate that behaviourally driven closed-loop beta tACS modulates post-movement beta ERS in a temporally specific manner, enhancing beta power mainly within the stimulation-aligned window.

### Beta tACS increases retention of a visuomotor adaptation task

**Fig. 4:**
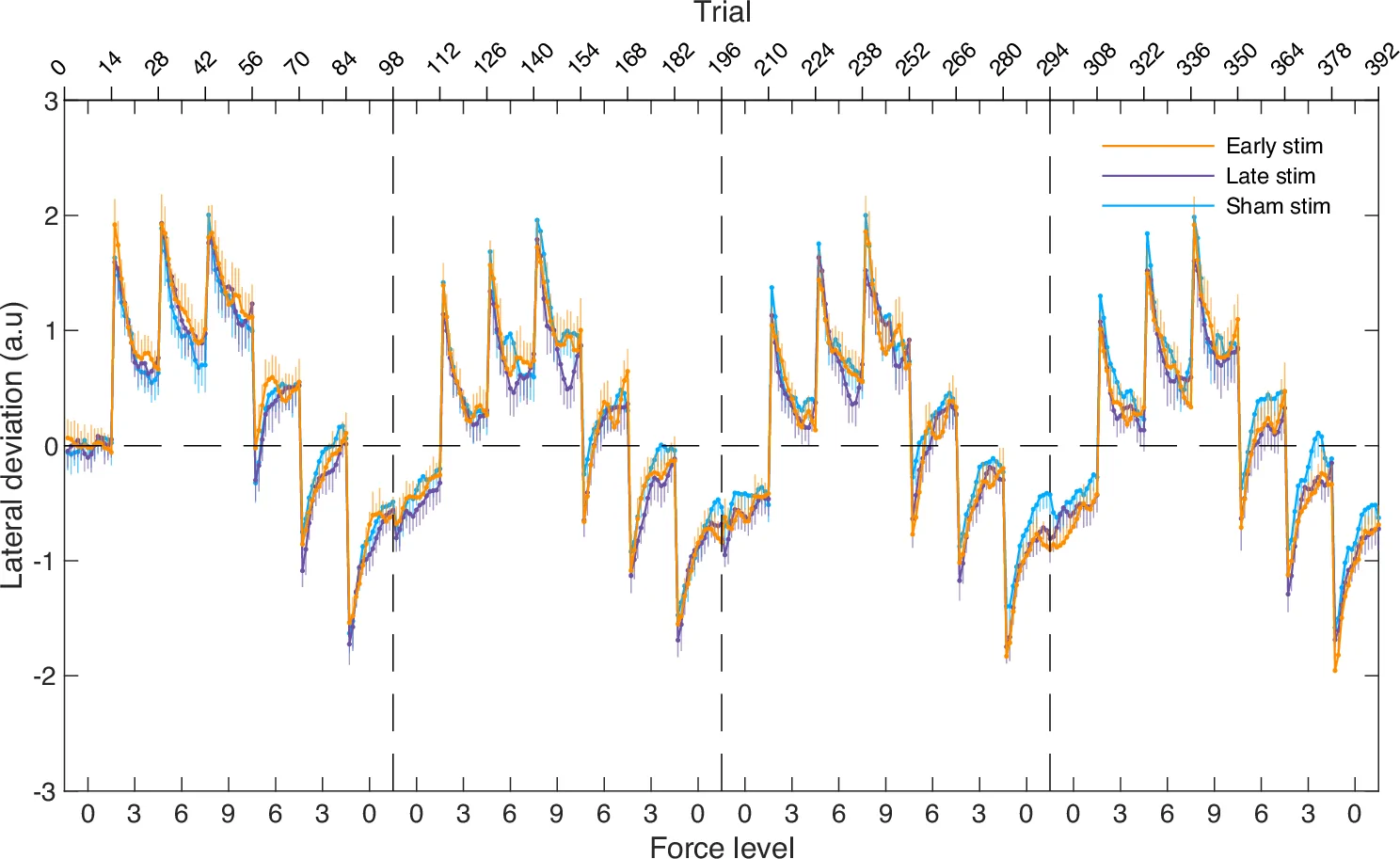
Trial-by-trial motor performance across stimulation conditions (*n* = 24 participants). Mean lateral deviation (±s.e.m) across all participants during the adaptation phase is shown across trials, as the force field changed in the sequence: 0-3-6-9-6-3-0, repeated for four blocks. Each force magnitude was applied for 14 consecutive trials. Positive values reflect deviations in the direction of the applied force (e.g. leftward deviation in response to a right-to-left force field). Negative values reflect deviations opposite to the force direction. Deviations were baseline-corrected using the mean from the initial zero-force trials of the first block.

Having established that behaviourally driven closed-loop tACS leads to an increase in beta ERS, we next wished to investigate whether this increase in activity drove changes in behaviour. As hypothesised, when the force field perturbation was applied, participants’ movement trajectories exhibited a systematic deviation from the straight line between the start point and the target (Figs. 1b and 4). An increase in lateral deviation was observed immediately upon transitioning to a different force magnitude and gradually decreased over subsequent trials within the same magnitude of applied force (Fig. 4).

**Fig. 5:**
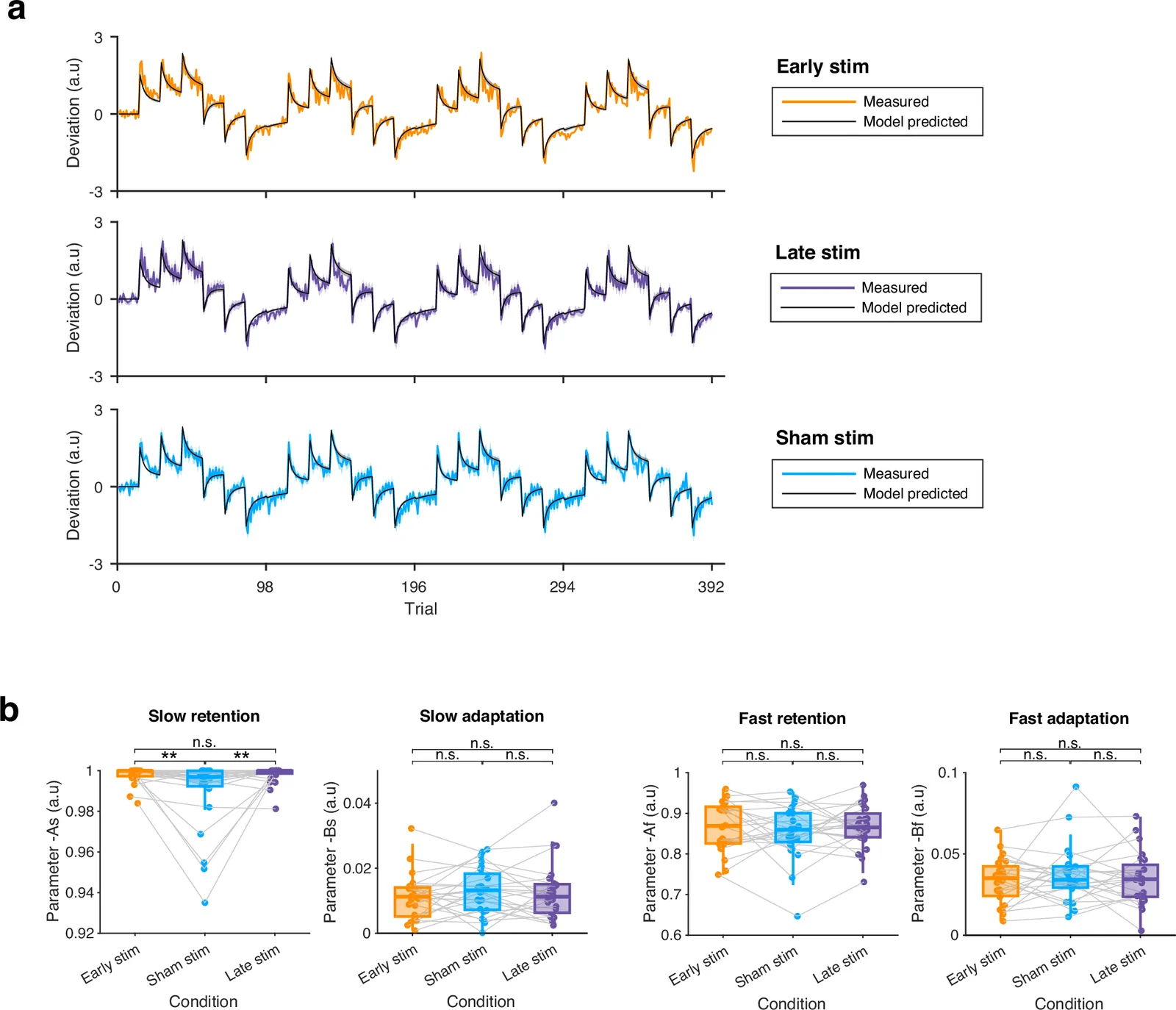
Two-state model fitting of motor adaptation across stimulation conditions. **a** Measured (coloured) and two-state model-simulated (black) trial-by-trial deviations (mean ±  s.e.m) during the motor adaptation task for early (orange), late (purple) and sham (blue) stimulation conditions (*n* = 24 participants). **b** Estimated model parameters across conditions: slow retention factor (*A*_s_), slow learning rate (*B*_s_), fast retention factor (*A*_f_), fast learning rate (*B*_f_) (*n* = 24 per condition; repeated measures). Each dot represents an individual participant; grey lines link participant data across conditions. The horizontal line within each box indicates the median; the bottom and top edges of the box indicate the first and third quartiles, respectively; and the whiskers indicate the most extreme values within 1.5 times the IQR. The slow retention parameter (*A*_s_) was significantly increased in both early and late stimulation conditions compared to sham, indicating enhanced retention (early versus sham, *t* = 2.850, *p* = 0.009, FDR-corrected; late versus sham, *t* = 2.955, *p* = 0.009, FDR-corrected). No significant differences were observed in the other parameters (all *p* > 0.05). n.s. non-significant, **p < 0.01. a.u. arbitrary units, IQR interquartile range.

Improvements on visuomotor adaptation tasks rely on two distinct behavioural processes: an adaptation to the new perturbation, which is thought to primarily be encoded in the cerebellum, and the retention of that new visuomotor transform, which is dependent on cortical regions^22^. We therefore fitted a state-of-the-art, two-state model to capture the multiple processes^23^. The model fitted well across all stimulation conditions (early: RMSE = 0.88 ± 0.03; late: RMSE = 0.86 ± 0.03; sham: RMSE = 0.86 ± 0.02; Fig. 5a). The estimated parameters revealed a significant effect of stimulation condition on the retention rate of the slow process (*A*_s_, *χ*^2^(2) = 11.733, *p* = 0.003). *A*_s_ was significantly higher during early tACS compared to sham (*t*(50.1) = 2.850, *p* = 0.009, FDR-corrected), reflecting a tACS-induced enhanced retention of the slow process. A_s_ was also higher during late tACS compared to sham (*t*(50.1) = 2.955, *p* = 0.009, FDR-corrected). No difference was found between early and late tACS (*t*(50.1) = −0.105, *p* = 0.917, FDR-corrected; Fig. 5b). No significant differences between stimulation conditions were found for other model parameters (*p* > 0.05 for *A*_f_, *B*_f_ and *B*_s_; Fig. 5b), indicating that the effect of stimulation was specific to slow retention.

**Fig. 6:**
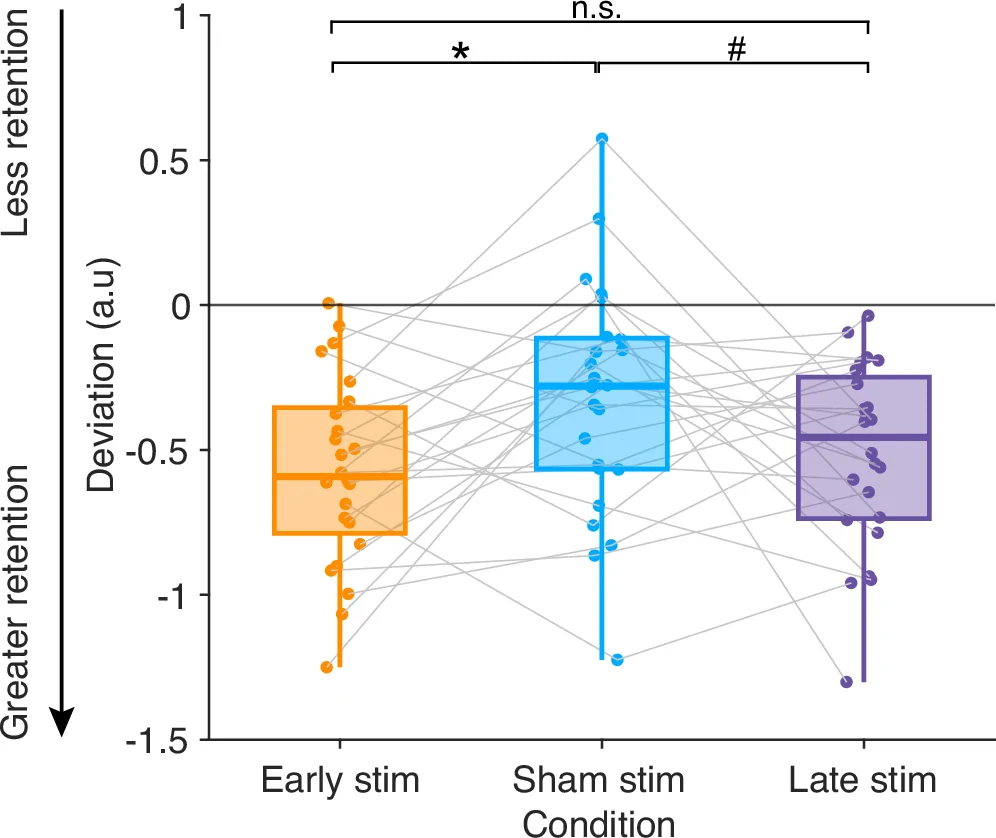
Measured retention across stimulation conditions. Motor performance measured as lateral deviation during the retention phase, shown for early stimulation (orange), sham (blue) and late stimulation (purple) conditions (*n* = 24 participants per condition; repeated measures). More negative values indicate greater retention of the adapted state. Each dot represents an individual participant; grey lines link participant data across conditions. The horizontal line within each box indicates the median; the bottom and top edges of the box indicate the first and third quartiles, respectively; and the whiskers indicate the most extreme values within 1.5 times the IQR. Early stimulation resulted in significantly greater retention compared to sham, and a marginal difference was also observed between late and sham stimulation (early versus sham, *t* = −2.622, *p* = 0.035, FDR-corrected; late versus sham, *t* = −2.006, *p* = 0.075, FDR-corrected). ^#^*p* < 0.1, *p < 0.05. a.u. arbitrary units, IQR interquartile range.

To confirm these findings from our model fit, we also directly quantified retention in no-vision retention trials. Similarly to the model fit results, a significant effect of stimulation condition was observed (*χ*^2^(2) = 7.846, *p* = 0.020). During early tACS, participants showed more negative lateral deviation, compared to the sham condition (*t*(50.1) = − 2.622, *p* = 0.035, FDR-corrected), reflecting stronger retention of the previously adapted motor output. A marginal difference was observed between late stimulation and sham (*t*(50.1) = − 2.006, *p* = 0.075, FDR-corrected; Fig. 6). No significant differences were observed between early and late stimulation (*t*(50.1) = − 0.616, *p* = 0.541, FDR-corrected). These results are consistent with the model-derived estimates, suggesting that closed-loop beta tACS enhanced both model-predicted retention and directly quantified retention.

### Enhanced beta ERS is associated with greater retention

We have shown that tACS leads to an increase in beta ERS amplitude and to a specific improvement in retention. This stimulation effect was further verified by a leave-one-participant-out sensitivity analysis (*p* ranges: ERS at early window: 0.003–0.025; ERS at late window: 0.001–0.009; *A*_s_: 0.002–0.012), by refitting the mixed-effects model 24 times, each time excluding one participant. Finally, we wanted to determine whether these neural and behavioural effects were related. To address this, we included beta ERS power, computed across the broad post-movement window spanning both the early and late stimulation periods, as a predictor of retention in a linear mixed-effects model. Higher beta ERS was significantly associated with greater *A*_s_ values (*β* = 0.007, *p* = 0.045), suggesting that enhanced post-movement beta ERS correlates with increased retention of the novel visuomotor transform. However, the beta ERS × stimulation condition interaction was not significant (*χ*^2^(2) = 4.388, *p* = 0.112), indicating that the slope relating beta ERS to *A*_s_ did not differ significantly between conditions. Furthermore, no significant correlation was observed between stimulation-induced changes in beta ERS and *A*_s_ (*β* = 0.0005, *p* = 0.868), indicating that inter-individual variation in ERS modulation did not linearly predict the magnitude of behavioural benefit in this sample.

### Blinding of stimulation conditions was effective

Lastly, we wanted to ensure that neither the participants nor the experimenter were able to distinguish between the three stimulation conditions. There was no evidence that reporting was better than chance (participants: *p* = 0.266; experimenter: *p* = 0.205), with 16 out of 24 participants reporting the sham condition as active and the experimenter frequently reporting 'don’t know' (Supplementary Fig. S3a, b), indicating successful participant blinding. No serious adverse events were reported. The most frequently reported sensations were mild tingling, itching and sleepiness. There were no significant differences in the frequency of reported sensations across stimulation conditions (all *p* > 0.05, Supplementary Fig. S3c).

## Discussion

Here, we have developed and implemented a behaviourally triggered closed-loop tACS approach, which is capable of specifically increasing the post-movement-related beta ERS, with an associated increase in retention, compared with sham stimulation.

### Behaviourally triggered closed-loop beta tACS increases movement-related beta ERS

We have shown that our behaviourally triggered beta tACS increases movement-related beta ERS. Importantly, our EEG analyses only included trials without concurrent stimulation, ensuring that the observed increases in beta power reflect changes in brain activity without being contaminated by stimulation artefacts. While tACS commonly induces entrainment during active stimulation ('online' effects) and can outlast the stimulation, the temporally specific, offline enhancement of beta ERS observed here is more consistent with synaptic plasticity mechanisms, such as spike-timing-dependent plasticity^24,25^. This interpretation is supported by previous work using intermittent, brief alpha tACS, which found similar after-effects regardless of whether stimulation was phase-continuous or phase-discontinuous^26^. These findings suggest that the offline effects we observe here may be driven by synaptic plasticity rather than mere entrainment.

Previous work has demonstrated offline effects of continuous beta tACS, but the findings remain inconsistent^27–30^. Here, we show that even brief (2 s) trains of beta tACS can induce specific offline neural effects. This finding contrasts with studies using alpha tACS, where longer trains (8 s) but not shorter trains (3 s) induced offline effects^26^. We believe that this inconsistency is likely because the effectiveness of brief stimulation critically depends on the specific brain or behavioural state at the moment of stimulation^31,32^. For instance, even a 1 s tACS train during sleep can significantly modulate spindle activity^32^. Similarly, our closed-loop stimulation delivered immediately after movement offset, when the brain naturally exhibits increased beta activity, likely preferentially enhances cortico-subcortical motor plasticity and reinforces functionally relevant motor network activity.

Behaviourally triggered closed-loop beta tACS specifically improves cortically dependent behaviour performance on a visuomotor adaptation task. This task relies on two distinct but concurrent behavioural processes, requiring the participant both to adapt to the new perturbation, which is primarily encoded in the cerebellum, and retain that new visuomotor transform, a cortically-dependent process^22,33,34^. In line with previous brain stimulation studies, we therefore hypothesised that cortical tACS applied above M1 would lead to a specific effect on retention while having no effect on adaptation^22,35^. Our behavioural results support this hypothesis. We observed that beta tACS applied over M1 shortly after movement offset significantly improved retention. These findings align with existing evidence emphasising the crucial role of M1 in motor retention. For example, studies using transcranial magnetic stimulation (TMS) have shown that reducing M1 excitability shortly after adaptation disrupts retention, suggesting M1 is vital for early memory consolidation^36,37^. Conversely, studies indicate that enhancing M1 excitability utilising anodal transcranial direct current stimulation immediately after adaptation improves retention^22,35^. Our findings extend these studies by demonstrating that short, behaviourally triggered bursts of beta tACS delivered to M1 can also enhance retention. Owing to the short, but neurophysiologically relevant, nature of the stimulation, the same total duration of stimulation can be applied over more movement repetitions, compared to conventional continuous stimulation.

Indeed, previous work highlights a temporally sensitive window immediately after training, during which the motor cortex is particularly receptive to neuromodulation. For instance, single-pulse TMS applied immediately after trial completion disrupts retention, whereas stimulation applied slightly later (700 ms post-trial) does not have the same disruptive effect^37^. Our data suggest that while immediate post-movement stimulation (i.e. early stimulation) strongly enhances retention, stimulation applied at a later period (i.e. late stimulation) also has a marginal benefit on behaviour. A plausible explanation for this could be that our task led to a relatively prolonged post-movement beta ERS compared with hand-held joystick tasks. Thus, even stimulation applied slightly later may interact beneficially with ongoing neural processes related to motor retention.

However, we only assessed retention during a single session and did not examine longer-term behavioural effects (e.g. 24-h afterwards). Therefore, it remains to be tested whether the observed improvements in retention would persist over time and whether repeated stimulation sessions could enhance or sustain the benefits.

### Movement-related beta ERS amplitude is correlated with cortically dependent retention

In our study, applying beta tACS immediately after the movement increased post-movement beta ERS, and this increase was associated with a larger retention factor in the slow process. Our results align with recent findings demonstrating that 20 Hz tACS over the motor cortex after training enhances motor retention of performance measured after 24-h and 7-days compared to sham stimulation^38^. Similarly, previous studies have shown that beta-tACS increases corticomuscular coherence, which positively correlates with improved motor retention^18^. The relationship between beta ERS and retention was also observed in observational studies. For example, healthy individuals who exhibit larger increases in beta ERS during practice typically show stronger retention of motor performance. In contrast, patients with Parkinson’s disease show smaller beta ERS and impaired motor retention^39^.

Mechanistically, a stronger beta ERS may indicate a brain state that supports memory retention by reinforcing the current motor plan and reducing the need for further corrections. Previous work has proposed that beta activity increases when the brain is trying to maintain a stable motor state^40^. During learning, a strong beta ERS after movement indicates more confidence in the motor output or the internal forward model. Conversely, a weak beta rebound might represent a need for further updating or correction^6^. Therefore, stronger beta ERS following beta-tACS was associated with greater retention of motor output.

However, since EEG analysis was restricted to unstimulated trials, the observed ERS enhancement reflects a between-trial aftereffect rather than direct online modulation of beta-tACS. This raises the question of whether the behavioural improvement is directly caused by the enhanced ERS or by other mechanisms induced by the beta stimulation. One possibility is that tACS altered the baseline neural state, such as general cortical excitability. However, our analysis of baseline beta power showed no differences between stimulation conditions, suggesting that the effect is not due to a global shift in baseline activity. Nevertheless, we cannot rule out that the stimulation modulates other unidentified neural signatures (e.g. brain connectivity) that concurrently improved behaviour and enhanced ERS. This interpretation is further supported by the finding that changes in beta ERS did not significantly predict changes in *A*_s_. In addition, the mixed-effects analysis did not provide evidence that stimulation reliably altered the relationship between beta ERS and *A*_s_.

### Towards behaviourally triggered closed-loop stimulation for real-world settings

We have demonstrated the ability of closed-loop stimulation to specifically interact with key neural dynamics, resulting in behavioural improvement. This has many potential applications as a putative therapeutic tool, but to be successful, it needs to be practicable to achieve. Conventional closed-loop brain stimulation approaches typically rely on continuous EEG monitoring, real-time neural decoding and complex artefact removal^7,19,41–43^, limiting their utility for clinical and practical applications, particularly in noisy or mobile settings. Our approach simplifies these challenges by using wearable motion sensors and mobile EEG, designed to directly translate into the home setting if required. Triggering the tACS via behaviour means that we can accurately target behaviourally-relevant neural activity and avoid the need for continuous EEG monitoring and artefact correction. However, while behavioural triggering increases the likelihood of stimulating during the intended post-movement beta state, ERS onset and duration can vary across participants and trials, such that alignment with the endogenous beta state is approximate rather than exact on every trial. This variability further motivates future approaches that combine behavioural and neural signals to guide closed-loop stimulation.

This approach is also highly efficient with a total stimulation duration per session of ~8.7 min. This duration is substantially less than that of conventional tACS protocols lasting 20–30 min^44–48^, but is still capable of robustly enhancing post-movement beta ERS, thereby reducing the potential risks and burden to the participant. Importantly, although age did not significantly influence any behavioural or neural outcomes in our study (all *p* > 0.05), our sample was restricted to young adults. Therefore, generalisation to older adults and potential age-related modulation of the effects require further study. Furthermore, although the present study targeted M1, other regions involved in motor control and learning, such as the premotor cortex, may also contribute to performance. Future work should compare M1-targeted stimulation with other motor cortical regions, such as premotor cortex, to determine the regional specificity of beta activity and its contribution to motor retention. We observed effects for both early and late stimulation, which may reflect a prolonged beta ERS such that both stimulation windows overlapped with the ERS period. This does not rule out the temporal specificity of beta-tACS; future studies should place stimulation clearly outside the ERS window (e.g. using a longer delay after movement offset) to more directly test temporal dependence. In addition, future work should directly compare closed-loop stimulation based on behaviour (as implemented here) with closed-loop stimulation based on neural activity, as well as with open-loop or continuous beta-tACS, to determine whether closed-loop delivery provides unique advantages beyond timing relative to endogenous beta activity.

In conclusion, we designed and implemented a portable, closed-loop neuromodulation system using motion sensor-based triggering of beta-frequency tACS. We demonstrated that behaviourally-triggered closed-loop tACS can effectively modulate specific aspects of neural activity and improve retention on a visuomotor adaptation task. This technique, therefore, opens a putative therapeutic route to improve motor function in clinical populations characterised by impaired movement-related beta activity, such as people with Parkinson’s disease or stroke.

## Methods

### Participants

Twenty-seven healthy participants (18–35 years old, 17 females) were recruited for this study. All participants were right-handed according to the Edinburgh Handedness Inventory and had normal or corrected-to-normal vision^49^. All participants fulfilled the following inclusion criteria: (1) no history or current neurological or psychiatric disease; (2) no impairment of upper limb function; and (3) no use of drugs affecting the central nervous system. Safety screening questionnaires for brain stimulation were completed prior to the experiment. Written informed consent was obtained from all participants in accordance with the Declaration of Helsinki. This study was approved by the Central University Research Ethics Committee, University of Oxford (Ethics Reference: R81071/RE003). Three participants withdrew before completing all experimental sessions (one reported scalp skin intolerance following the second experimental session, and two dropped out due to personal reasons). Twenty-four participants completed the experiment and were included in the analyses. Participants were compensated GBP 10 per hour.

### Study design

This within-subject, double-blinded, sham-controlled study included three sessions, with the only difference between sessions being the stimulation condition. The order of stimulation was counterbalanced across participants using a randomisation code. Both participants and the experimenter, interacting with the participants, were blinded to the stimulation allocation. A second experimenter, responsible for technical implementation, was not involved in participant interaction and did not disclose stimulation details. Sessions were separated by at least seven days (median 7 days; interquartile range [IQR] 7–9.5). To assess blinding efficacy, immediately after each session, both the participant and the interacting experimenter completed a brief blinding questionnaire indicating their belief about whether the session involved active or sham stimulation using a 5-point scale (1 = strongly active, 2 = somewhat active, 3 = somewhat sham, 4 = strongly sham, 5 = don’t know).

### Motor adaptation task and behavioural data acquisition using Kinarm

In each session, participants performed a target-reaching task with their right hand using a Kinarm END-point robotic system (Kinarm, Kingston, Canada). Participants were seated in a height-adjustable chair in front of the Kinarm and faced a horizontal mirror that reflected visual stimuli from a downward-facing monitor. The right Kinarm handle was displayed on the screen as a white circle (radius: 0.5 cm). Handle position (x, y) and velocity in the horizontal plane were recorded at a sampling rate of 1000 Hz using the Kinarm system.

The task required participants to use the right Kinarm handle to make straight, planar reaching movements between two target points spaced 15 cm apart, alternating between outward (away from the body) and inward (toward the body) directions. At the start of each trial, participants positioned the handle at the start point. Once the handle was held at this position, the target turned yellow, indicating a go cue. Participants were instructed to move as quickly and accurately as possible to the target. Upon reaching it, the target turned purple and then became the start point for the next trial. This ensured an alternation of inward and outward movements. An inter-trial interval of 5 s was used to quantify the post-movement beta ERS.

The movement task in each session consisted of three sequential phases: practice, adaptation and retention. During practice, participants completed 14 trials without any force field perturbation, and feedback was given if the movement was not performed fast enough. The adaptation phase included a total of 392 trials, divided into four blocks of 98 trials each. A velocity-dependent lateral force field was introduced during this phase, applying force along the x-axis (perpendicular to movement direction). The magnitude of the force field changed every 14 trials following this sequence: 0, 3*f*, 6*f*, 9*f*, 6*f*, 3*f*, 0 (Fig. 1a), see section ‘Two-state model fitting’ for more details. Participants were not informed about the presence of the force perturbations. The direction of the force perturbation (leftward or rightward) remained constant within a session but varied across sessions. The order of the force direction across three sessions (i.e. left-right-left or right-left-right) was counterbalanced across participants and stimulation conditions. Both during practice and adaptation phases, continuous visual feedback of the handle position was provided. Immediately after the adaptation phase, a retention phase of 14 trials was conducted. In these trials, no force was applied, and the visual feedback of the handle was removed during movement. This phase allowed assessment of the retention of the adapted motor response in the absence of visual feedback.

### Closed-loop transcranial alternating current stimulation (tACS)

Kinematic data were also acquired using a single VIVE Tracker 3.0 and two HTC VIVE Base Stations (HTC, Taiwan). Kinematic data from the VIVE Tracker were used to trigger the brain stimulation. We opted to trigger brain stimulation from the kinematic data obtained from the VIVE Tracker rather than the Kinarm, as the VIVE Tracker is more affordable and portable, allowing for future studies outside the laboratory. The VIVE Tracker was rigidly attached to the Kinarm handle and remained in place throughout the entire study. The coordinate systems of the Kinarm robot and the VIVE Tracker were aligned. VIVE Tracker positional data were wirelessly streamed via a VIVE Dongle at a sampling rate of 90 Hz. A custom C++ application (ViveTrackQt) continuously recorded the VIVE Tracker’s position and interfaced in real time with a MATLAB script via a TCP/IP connection. When the VIVE Tracker (attached to the Kinarm handle) had entered the target area (defined as time *t*_₀_) and remained continuously within this area for at least 150 ms (defined as time *t*_1_, Fig. 1c), stimulation was triggered.

TACS was applied during the adaptation phase but not the practice or retention phases. tACS was delivered using a Starstim 8 system (Neuroelectrics, Barcelona, Spain). Two 1π cm^2^ Ag/AgCl electrodes (Starstim NG Pistim) were integrated into an EEG cap, with the stimulation electrode placed over the left motor cortex (C3) and the return electrode positioned at Pz according to the standard 10–20 system. Stimulation was applied at 20 Hz, with the current amplitude of ±1 mA (2 mA peak-to-peak). Electrode impedance was maintained below 10 kΩ throughout each session. To estimate the spatial distribution and magnitude of the induced electric field in the brain, we performed simulations using the SimNIBS software toolbox (version 4.5.0) with the built-in head model Ernie^50^. Electric field simulations confirmed that the peak field intensity occurred in the left sensorimotor cortex (Supplementary Fig. S5), while the stimulation also engaged the adjacent dorsal premotor cortex.

Three distinct stimulation conditions were used. In the early stimulation condition, 20 Hz tACS was delivered immediately following this 150 ms hold period (*t*_1_) and lasted for 2 s. In the late stimulation condition, stimulation was delivered 2.5 s after *t*_1_ and also lasted 2 s (Fig. 1c and Supplementary Fig. S6). In the sham condition, stimulation was similarly triggered 2.5 s after *t*_1_ but lasted only 100 ms. All stimulation trains included 50 ms ramp-up and ramp-down periods. tACS was applied in four out of every six trials, with the remaining two trials left stimulation-free, and thus artefact-free for EEG data analysis. Therefore, for each force level, 18–20 of the 56 trials were stimulation-free, resulting in a total of 130–132 stimulation-free trials per session (Supplementary Fig. S4).

### Behavioural data analysis

The velocity signals recorded from the Kinarm system were low-pass filtered using a fourth-order Butterworth filter with a 30 Hz cutoff. Movement onset was defined as the time point when velocity exceeded a threshold, calculated as the mean plus three standard deviations (SD) of the velocity during the 'rest' period and remained above this threshold for at least 100 ms. Similarly, movement offset was defined as the time before the velocity fell below this threshold and remained there for at least 100 ms. Onset and offset were identified within each trial. The interval from movement onset to movement offset was defined as the movement period for each trial.

Movement performance was quantified by the maximum lateral deviation, defined as the peak perpendicular distance between the Kinarm handle trajectory and the straight line connecting the start point and target. This was achieved by using the *findpeaks* function in MATLAB, with a minimum peak width of 100 ms. The deviation sign was defined relative to the direction of the applied force field: deviations toward the force direction were considered positive, while deviations opposite the force direction were negative. Although no force was applied during the retention phase, the deviation sign was defined relative to the force direction used in the previous adaptation phase.

To account for individual baseline biases, lateral deviation values were baseline-corrected by subtracting the mean deviation during the initial zero-force trials of the first block (Fig. 1a). Measured retention was defined as the mean lateral deviation across all trials in the retention phase.

### Two-state model fitting

To further characterise motor adaptation and retention processes, we fitted a two-state model to the trial-by-trial lateral deviation data in the adaptation phase^23^. In this model, the motor output ${\rm X}\left(n\right)$ on trial n, is the sum of a fast process ${{\rm X}}_{f}(n)$ and a slow process ${{\rm X}}_{{{{s}}}}(n)$. These two processes update independently based on the error $e\left(n-1\right)$ and motor output ${\rm X}(n-1)$ on the previous trial following:

1 $${\rm X}\left(n\right)={{\rm X}}_{f}\left(n\right)+{{\rm X}}_{s}\left(n\right),$$

2 $${{\rm X}}_{f}\left(n\right)={A}_{{{{f}}}}\cdot {{\rm X}}_{{{{f}}}}\left(n-1\right)+{B}_{{{{f}}}}\cdot e\left(n-1\right),$$

3 $${{\rm X}}_{{{s}}}\left(n\right)={A}_{{{s}}}\cdot {{\rm X}}_{{{s}}}\left(n-1\right)+{B}_{{{s}}}\cdot e\left(n-1\right)$$

Here, ${{\rm A}}_{{{f}}}$ and ${A}_{{{s}}}$ are retention factors, and ${B}_{{{f}}}$ and ${B}_{{{s}}}$ are learning (adaptation) rates for the fast and slow processes, respectively. All parameters were constrained to the interval [0, 1], with the additional constraints ${A}_{f} < {A}_{s}$ and ${B}_{{{f}}} > {B}_{{{s}}}$, reflecting the faster learning and lower retention of the fast process compared to the slow process.

The error $e\left(n\right)$, i.e. lateral deviation, was defined as the difference between the scaled force field and the motor output on each trial:

4 $$e\left(n\right)=k\cdot f\left(n\right)-X\left(n\right).$$

where $f\left(n\right)$ is the applied force magnitude on trial n and k is a free scaling parameter that maps the force magnitude to the measured lateral deviation. Force magnitudes were encoded as 0 (no force), 1 (force magnitude 9), and linearly interpolated for intermediate values (e.g. 1/3 for force magnitude 3 and 2/3 for magnitude 6).

Model fitting was performed by simulating the entire sequence of trials and minimising the mean squared errors between predicted and observed deviations using MATLAB’s *fmincon* function. Firstly, a group-level fit was conducted by concatenating all trials across participants and sessions, allowing estimation of five free parameters: ${A}_{f}$, ${B}_{{{{f}}}}$, ${A}_{{{{s}}}}$, ${B}_{{{{s}}}}$, and the scaling factor k. Secondly, individual-level fits were performed separately for each participant and session. For these individual fits, the four learning parameters (${A}_{f}$, ${B}_{{{{f}}}}$, ${A}_{{{{s}}}}$, ${B}_{{{{s}}}}$) were initialised using the group-level estimates, while the scaling factor k was held fixed to the group-level value. This procedure yielded participant- and session-specific estimates of the learning and retention. The goodness-of-fit was assessed using the root mean square error (RMSE).

### EEG recording and analysis

EEG data were continuously recorded during the movement task using a 64-channel wireless system (Smarting®, mBrainTrain®, Belgrade, Serbia) with an elastic EEG cap (EASYCAP, Herrsching, Germany). Electrodes were positioned according to a standard 10-20 system, with FPz as the ground electrode and FCz as the reference electrode. Electrode impedances were maintained below 10 kΩ throughout each session. EEG signals were digitized at a sampling rate of 250 Hz.

Data preprocessing was performed using EEGLAB (version 2024.0)^51^ and MATLAB (R2023a). Raw data were band-pass filtered between 1 and 45 Hz using a Butterworth Infinite Impulse Response (IIR) filter (zero phase shift, filter order: 6). The data were epoched from −2 to +7 s relative to movement onset. Epochs containing stimulation artefacts were rejected (remaining epochs, mean ± SD: 114 ± 19). Then, bad channels with a leptokurtic voltage distribution were replaced by interpolating between the surrounding electrodes (spherical spline interpolation; mean ± SD: 3 ± 3 channels). Next, independent component analysis (ICA) with the second-order blind identification algorithm was performed, and artefactual components were identified and discarded (mean ± SD: 13 ± 6 components) using the EEGLAB plugin ICLabel^52^.

Following artefact rejection, data were re-epoched for subsequent analyses: from −1.5 to +3 s relative to movement onset (for beta ERD) and from −1.5 to +5 s relative to target reached (for beta ERS). Time–frequency analysis was performed using Morlet wavelets in FieldTrip (version 20240614). Spectral power was estimated from 5 to 40 Hz (1 Hz steps), with a temporal resolution of 20 ms, using a fixed wavelet width of 9 cycles. Power was computed on a single-trial basis. Baseline correction was applied from −0.5 to −0.2 s relative to movement onset, and the results were converted to decibel (dB) units to normalise power.

Beta ERS was quantified within stimulation-aligned time windows: an 'early stimulation' window from 0 to 2 s and a 'late stimulation' window from 2.5 to 4.5 s relative to the time of movement offset (*t*_1_, see ‘Closed-loop tACS’). Beta ERD was computed from 0 to 1.5 s relative to movement onset. Analyses focused on 15–30 Hz and were restricted to electrodes over the contralateral sensorimotor cortex, including C1, C5, CP1, CP3, CP5, P1, P3 and P5, located between the two stimulation electrodes (C3 and Pz).

### Statistical analyses

Clusters showing beta power difference between stimulation conditions were identified using non-parametric cluster-based permutation tests (1000 permutations), with correction for multiple comparisons based on the temporal cluster size criterion^53^.

To assess the stimulation condition effects on behavioural and EEG outcomes, linear mixed-effects models (LMMs) were implemented using the lme4 package in R (version 4.5.2). Separate models were constructed for beta ERS power averaged from 0 to 4.5 s following movement offset, covering both the early and late stimulation time windows, beta ERD power, measured retention (lateral deviation during the retention phase), or model-derived parameters. Each model included stimulation condition (early, late, sham) as a fixed effect and participant as a random intercept. When a significant main effect was observed, post hoc pairwise comparisons were performed with false discovery rate (FDR) correction. Additional LMMs were used to test whether model parameters could be predicted by beta ERS.

The effectiveness of participant and experimenter blinding was assessed using Fisher’s exact test to determine whether the distribution of perceived stimulation types differed across stimulation conditions. Similarly, sensations to stimulation (e.g. itching, tingling) were summarised descriptively, and condition differences were also evaluated using Fisher’s exact test.

### Reporting summary

Further information on research design is available in the Nature Portfolio Reporting Summary linked to this article.

## Supplementary information

Supplementary Information

Reporting Summary

Transparent Peer Review File

## Source data

Source Data

## Data Availability

The EEG data supporting the findings of this study have been deposited on OSF at https://doi.org/10.17605/OSF.IO/R6PHW. Source data are provided with this paper.

## Code availability

The code used to generate the figures and perform the reported analyses is available on OSF at https://doi.org/10.17605/OSF.IO/R6PHW.
