# Supplementary material for: Behaviourally driven closed-loop beta-tACS enhances beta activity and motor behaviour
Source: Nat Commun. 2026 Jul 21;17:8908. doi: 10.1038/s41467-026-75799-8 (PMC13503910; doi:10.1038/s41467-026-75799-8)
Supplement: Supplementary file 1 [file 41467_2026_75799_MOESM1_ESM.pdf]

Supplementary Information

Behaviourally driven closed-loop beta-tACS enhances beta activity and motor behaviour

Min Wu[1,2, *, #], Zeyu Xu[1,2, *], Melanie K Fleming[1], Nic Shackle[2], Lara Biller[1,2], Faye Tabone[1,2], Pei-Ling Wong[3], Caroline Nettekoven[4], Andrew Sharott[2], Catharina Zich[1,2, #], Charlotte J Stagg[1,2]

[#] **Correspondence:**

Dr Min Wu,

Oxford Centre for Integrative Neuroimaging, John Radcliffe Hospital, Oxford, OX3 9DU, UK

Email: min.wu@ndcn.ox.ac.uk

Dr Catharina Zich,

Oxford Centre for Integrative Neuroimaging, John Radcliffe Hospital, Oxford, OX3 9DU, UK

Email: catharina.zich@ndcn.ox.ac.uk

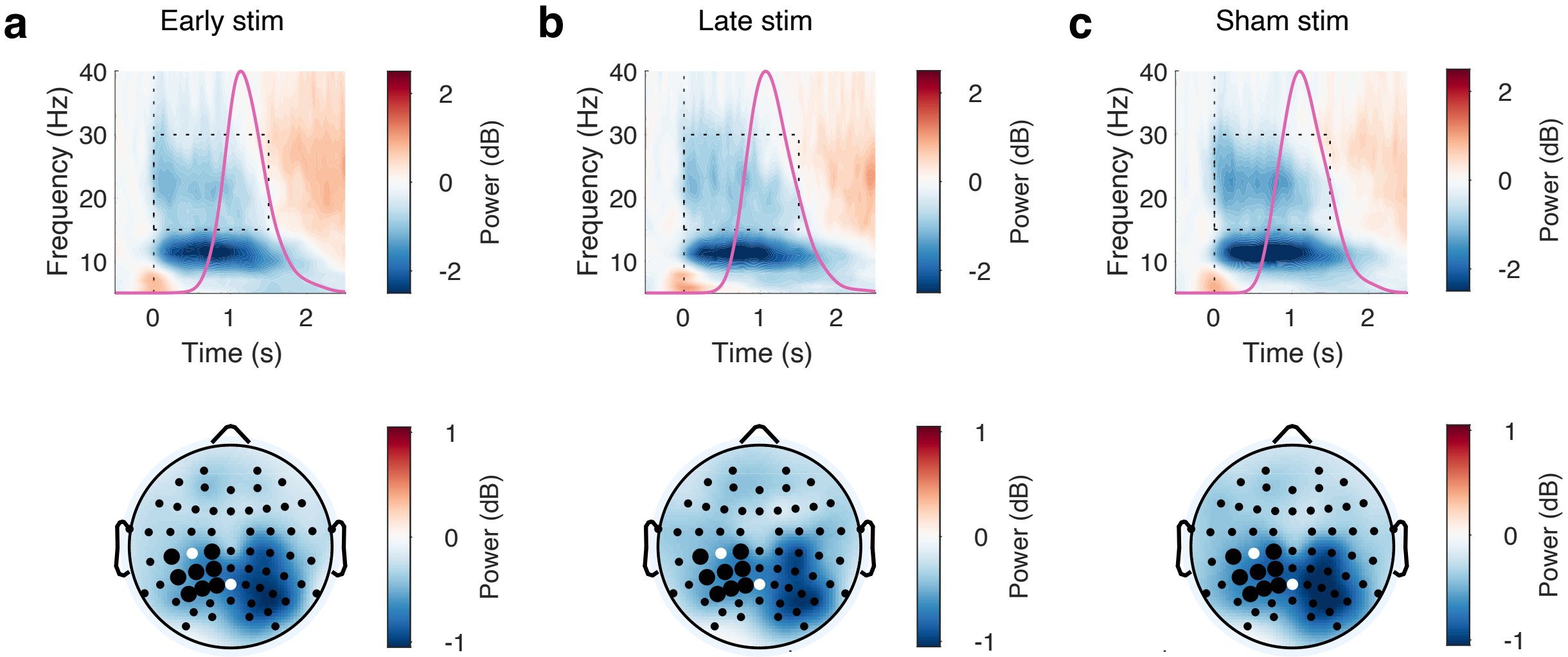

Figure S1. Time–frequency responses during movement across stimulation conditions (n = 24 participants). (a). Time-frequency response aligned to movement onset (t_0 = *0 s*) for the early stimulation condition. The power was averaged across sensors covering the contralateral sensorimotor area (highlighted in bold in bottom panel). The magenta curve shows the distribution of movement time across participants. The black rectangle indicates the time–frequency window used to quantify beta ERD (0–1.5 s, 15–30 Hz). The scalp topography shows bilateral distribution of beta ERD. Black dots mark electrodes in the predefined sensorimotor area; white dots indicate stimulation electrodes (C3 and Pz). **(b).** Similar as (a), for the late stimulation condition. **(c).** Similar as (a), for the sham stimulation condition.

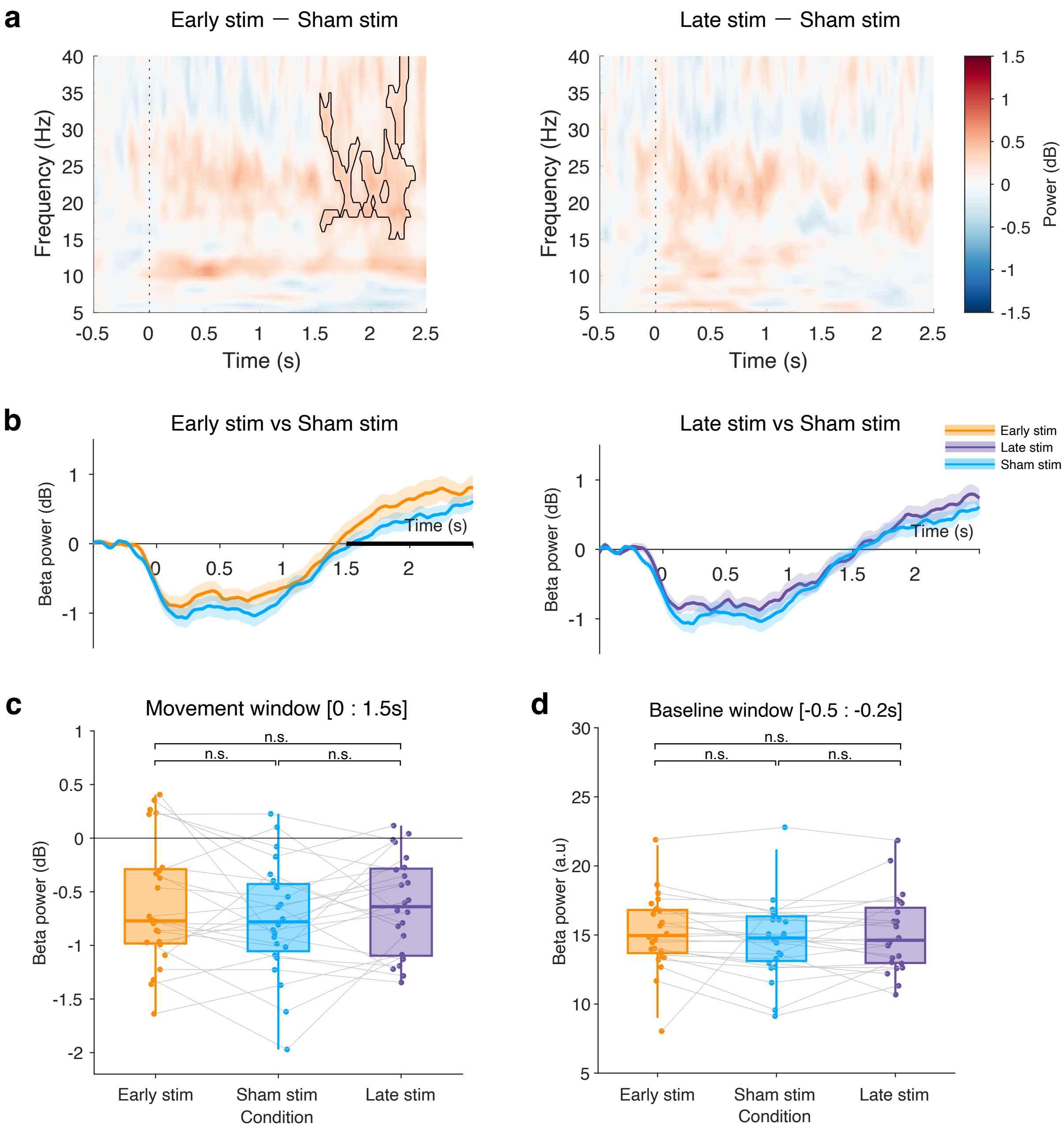

Figure S2. Modulation of beta activity during movement by stimulation conditions. (a). Time frequency response difference in the sensorimotor cortex between conditions (n = 24 participants). The black outlines indicate significant clusters with $p < 0.05$ by cluster-based permutation test (one-sided, 1000 permutations). **(b).** Time course of beta power (15–30 Hz) averaged over the contralateral sensorimotor area, aligned to movement onset (t_0 = 0 s). Left: comparison between early (orange) and sham stimulation (blue). Right: comparison between late (purple) and sham

(blue) stimulation. Black horizontal bars indicate time intervals with significant differences between conditions, determined by cluster-based permutation testing ($p < 0.05$). **(c).** Mean beta power extracted from movement onset to 1.5 s afterwards. **(d).** Mean beta power extracted from pre-movement baseline window over the contralateral sensorimotor area. Each dot represents an individual participant; grey lines link participant data across conditions. The horizontal line within each box indicates the median; the bottom and top edges of the box indicate the first and third quartiles, respectively; and the whiskers indicate the most extreme values within 1.5 times the interquartile range (IQR). No significant difference on beta ERD was observed across three stimulation conditions.

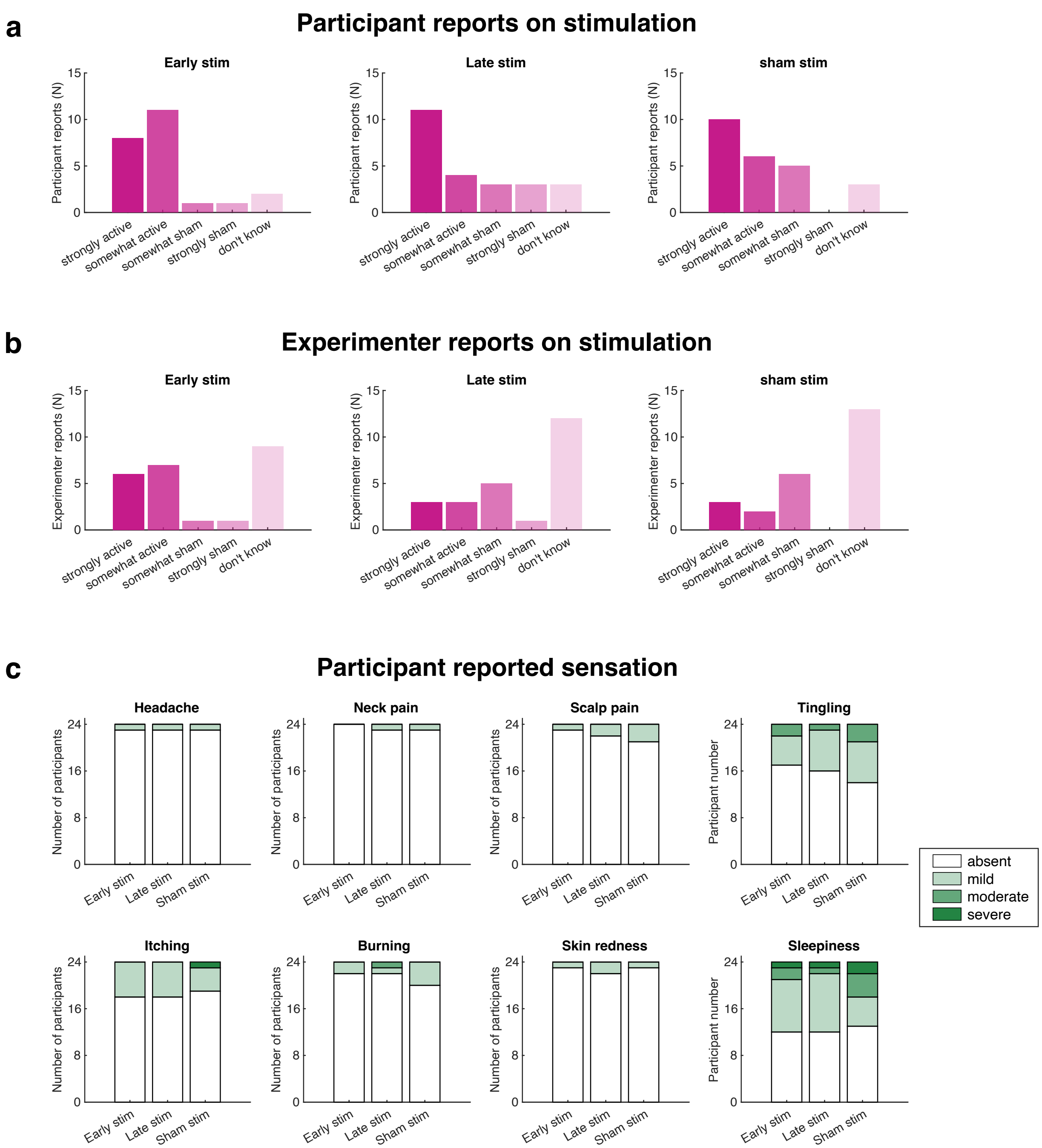

Figure S3. Blinding effectiveness and reported sensations across stimulation conditions. (a-b). Distribution of reported stimulation type from participants and experimenter in early, late, and sham stimulation conditions. Bars show the number of participants / experimenter reporting the stimulation as strongly active, somewhat active, somewhat sham, strongly sham, or don't know. **(c).** Participants' reported sensations during stimulation. Each panel corresponds to one

sensation type (e.g., tingling, itching, sleepiness). Stacked bars indicate the number of participants reporting each sensation at four severity levels: absent, mild, moderate, and severe.

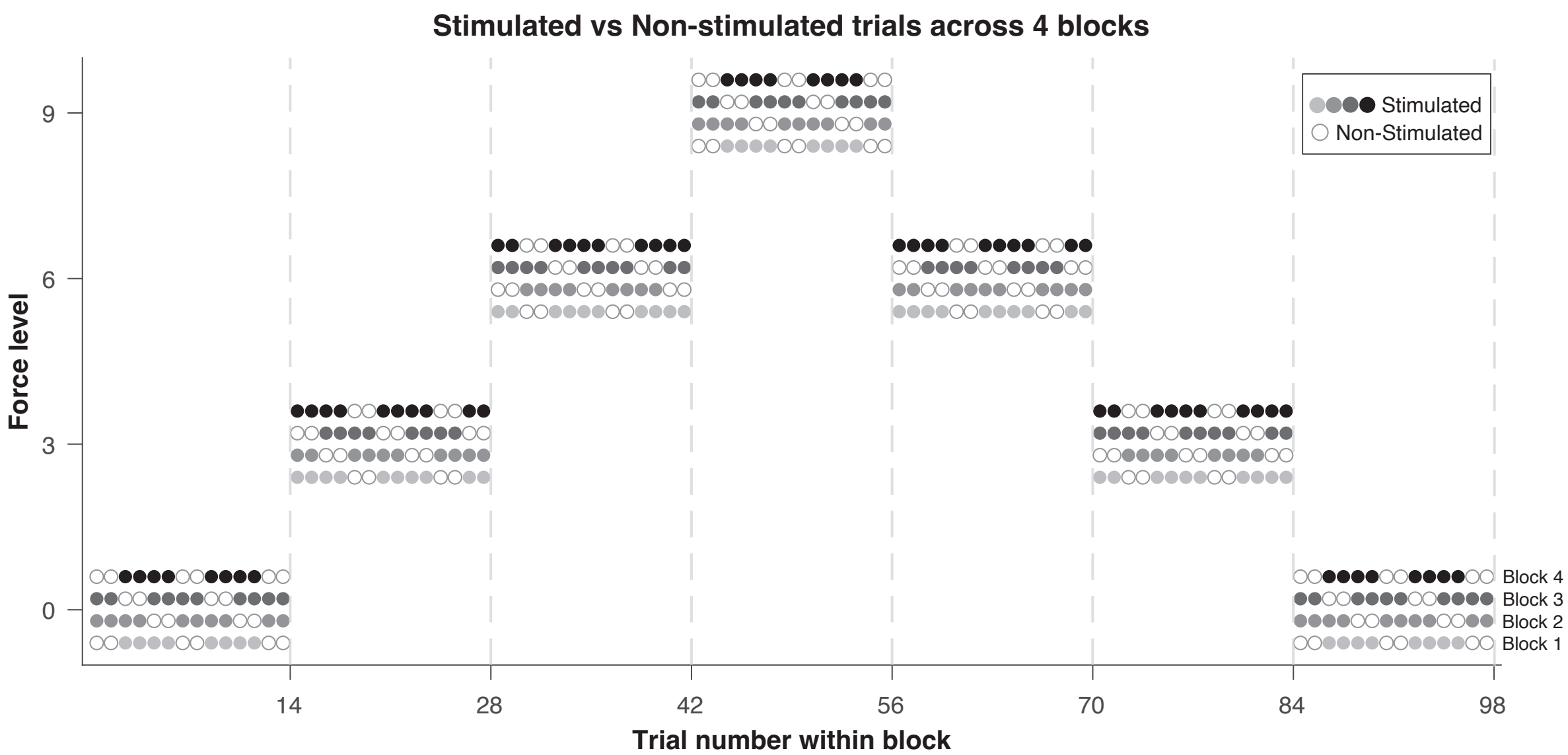

Figure S4. Distribution of stimulated and non-stimulated trials across force fields for a representative session. Each block consists of 98 trials (14 trials × 7 force levels: 0, 3, 6, 9, 6, 3, 0). The four repetitions of the block are stacked.

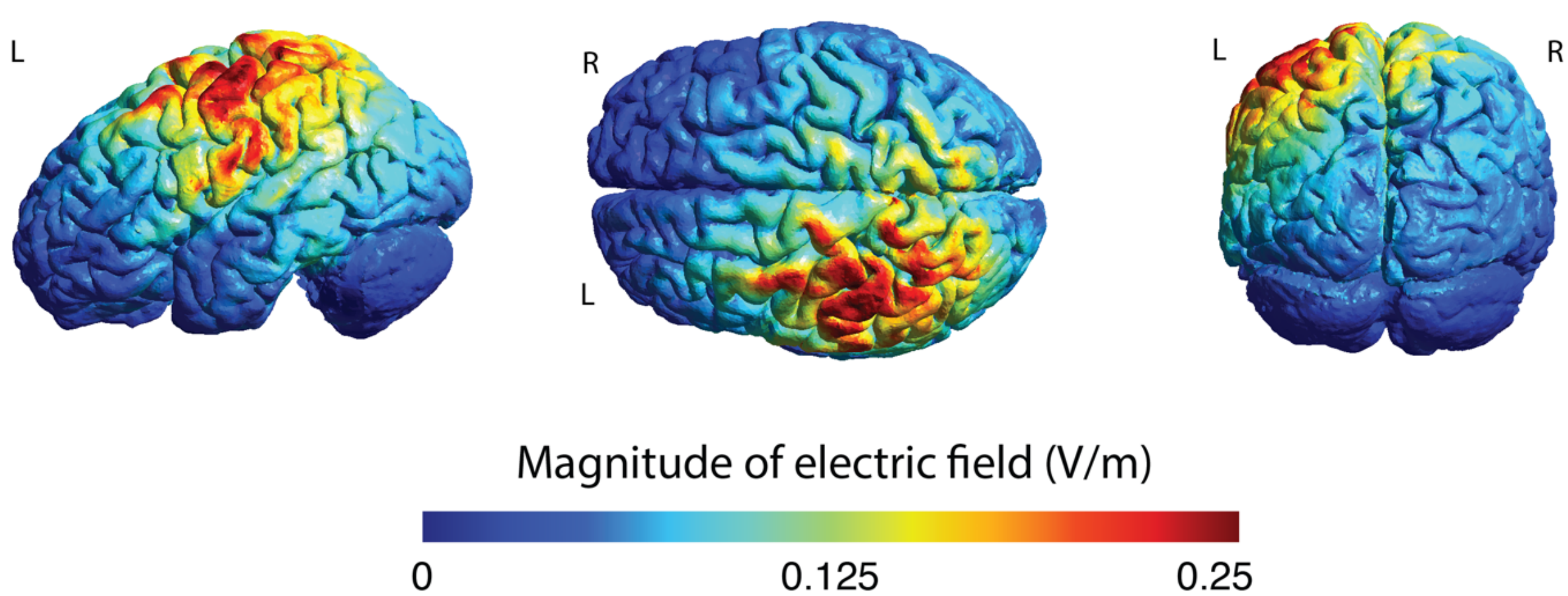

Figure S5. Electric field simulation of C3-Pz tACS.

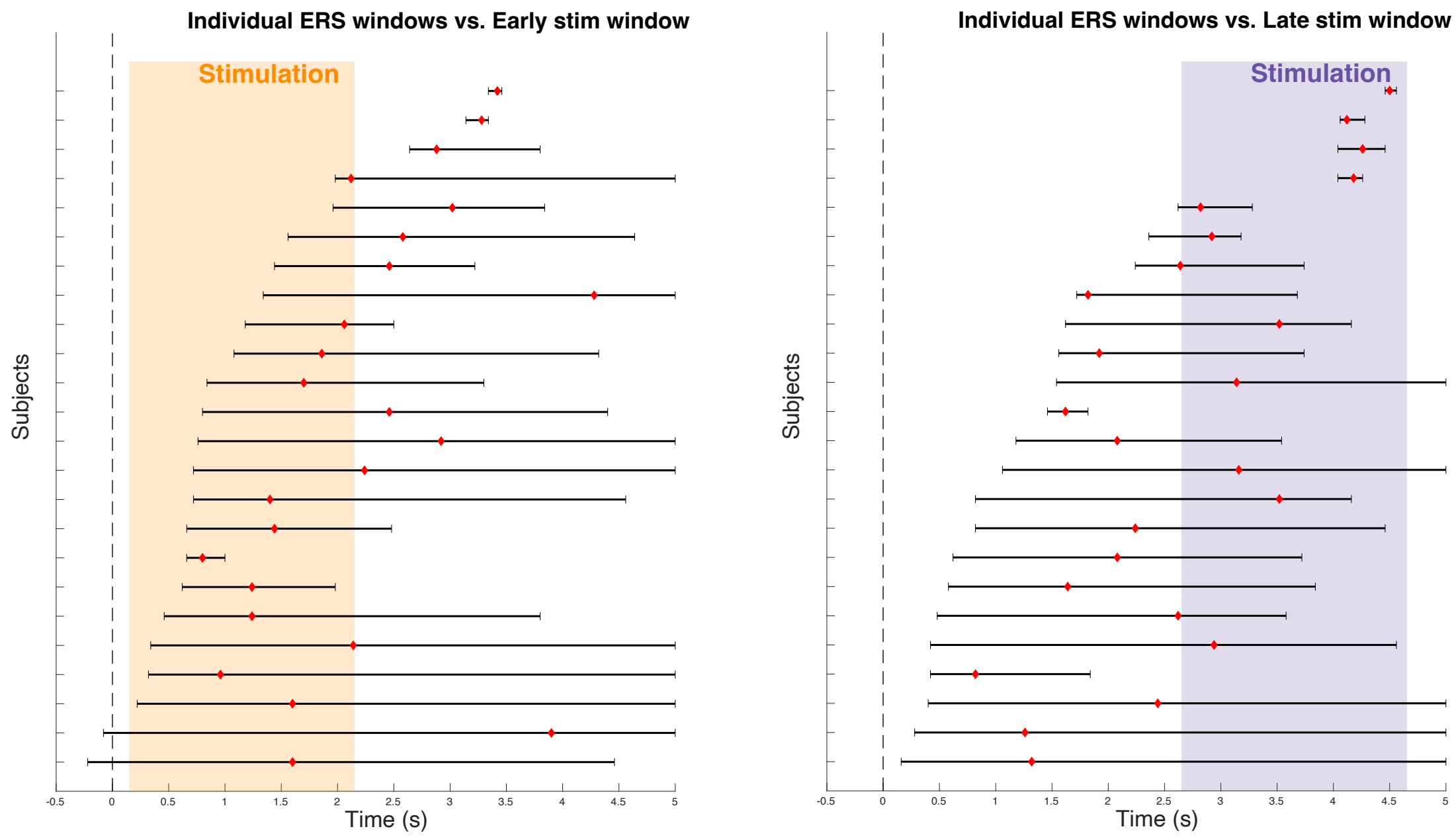

Figure S6. Individual post-movement beta ERS windows and stimulation windows. The black horizontal line indicates the ERS window (onset to offset), and the red dots denote the latency of the ERS peak.
