# Supplementary material for: Behaviourally driven closed-loop beta-tACS enhances beta activity and motor behaviour
Source: Nat Commun. 2026 Jul 21;17:8908. doi: 10.1038/s41467-026-75799-8 (PMC13503910; doi:10.1038/s41467-026-75799-8)
Supplement: Supplementary file 2 [file 41467_2026_75799_MOESM2_ESM.pdf]

nature portfolio

Corresponding author(s): Min Wu
Catharina Zich

Last updated by author(s): Jun 12, 2026

Reporting Summary

Nature Portfolio wishes to improve the reproducibility of the work that we publish. This form provides structure for consistency and transparency in reporting. For further information on Nature Portfolio policies, see our Editorial Policies and the Editorial Policy Checklist.

Statistics

For all statistical analyses, confirm that the following items are present in the figure legend, table legend, main text, or Methods section.

n/a	Confirmed	
☐	☒	The exact sample size (n) for each experimental group/condition, given as a discrete number and unit of measurement
☐	☒	A statement on whether measurements were taken from distinct samples or whether the same sample was measured repeatedly
☐	☒	The statistical test(s) used AND whether they are one- or two-sided<br>*Only common tests should be described solely by name; describe more complex techniques in the Methods section.*
☐	☒	A description of all covariates tested
☐	☒	A description of any assumptions or corrections, such as tests of normality and adjustment for multiple comparisons
☐	☒	A full description of the statistical parameters including central tendency (e.g. means) or other basic estimates (e.g. regression coefficient) AND variation (e.g. standard deviation) or associated estimates of uncertainty (e.g. confidence intervals)
☐	☒	For null hypothesis testing, the test statistic (e.g. F, t, r) with confidence intervals, effect sizes, degrees of freedom and P value noted<br>*Give P values as exact values whenever suitable.*
☒	☐	For Bayesian analysis, information on the choice of priors and Markov chain Monte Carlo settings
☒	☐	For hierarchical and complex designs, identification of the appropriate level for tests and full reporting of outcomes
☒	☐	Estimates of effect sizes (e.g. Cohen's d, Pearson's r), indicating how they were calculated

Our web collection on statistics for biologists contains articles on many of the points above.

Software and code

Policy information about availability of computer code

Data collection	Behavioural data were collected using the KINARM END-Point robotic system (Kinarm, Kingston, Canada) and VIVE Tracker 3.0 (HTC, Taiwan). A custom C++ application (ViveTrackQt) continuously recorded VIVE Tracker position and interfaced in real time with a MATLAB script via a TCP/IP connection. EEG data were acquired using a 64-channel wireless system (Smarting®, mBrainTrain®, Belgrade, Serbia) with an elastic EEG cap (EASYCAP, Herrsching, Germany).
Data analysis	EEG preprocessing and analysis were performed using MATLAB R2023a, EEGLAB v2024.0 and FieldTrip v20240614. Electric-field simulations were performed using SimNIBS v4.5.0. Statistical analyses were performed in R v4.5.2 using the lme4 package. Two-state model fitting and figure generation were performed using custom MATLAB scripts.

For manuscripts utilizing custom algorithms or software that are central to the research but not yet described in published literature, software must be made available to editors and reviewers. We strongly encourage code deposition in a community repository (e.g. GitHub). See the Nature Portfolio guidelines for submitting code & software for further information.

Data

Policy information about availability of data

All manuscripts must include a data availability statement. This statement should provide the following information, where applicable:

- Accession codes, unique identifiers, or web links for publicly available datasets
- A description of any restrictions on data availability
- For clinical datasets or third party data, please ensure that the statement adheres to our policy

Data and code are available on OSF at https://doi.org/10.17605/OSF.IO/R6PHW.

Research involving human participants, their data, or biological material

Policy information about studies with human participants or human data. See also policy information about sex, gender (identity/presentation), and sexual orientation and race, ethnicity and racism.

Reporting on sex and gender	Participants self-reported gender. The sample comprised 17 females and 7 males. Gender was not used as a stratification factor and no gender-specific analyses were performed because the study was not designed to assess gender differences.
Reporting on race, ethnicity, or other socially relevant groupings	Race and ethnicity data were not collected in this study.
Population characteristics	Healthy adults aged 18–35 years, with no self-reported neurological or psychiatric disorder.
Recruitment	Recruitment was conducted in accordance with the approved ethics application via word of mouth, departmental mailing lists and posters. We endeavoured to reduce any recruitment bias by keeping the inclusion criteria for the study as open as possible. Participants were compensated GBP 10 per hour.
Ethics oversight	The study was approved by the Central University Research Ethics Committee, University of Oxford (Ethics Reference: R81071/RE003). All participants provided written informed consent.

Note that full information on the approval of the study protocol must also be provided in the manuscript.

Field-specific reporting

Please select the one below that is the best fit for your research. If you are not sure, read the appropriate sections before making your selection.

☒ Life sciences ☐ Behavioural & social sciences ☐ Ecological, evolutionary & environmental sciences

For a reference copy of the document with all sections, see nature.com/documents/nr-reporting-summary-flat.pdf

Life sciences study design

All studies must disclose on these points even when the disclosure is negative.

Sample size	A total of N = 24 participants completed the study, each attending three sessions (within-subject design). A formal a priori power calculation was not performed because no prior studies with directly comparable outcomes were available. Robustness of the effects was evaluated using leave-one-subject-out analyses.
Data exclusions	Twenty-seven participants were recruited. Three withdrew before completing all sessions: one because of scalp intolerance after the second session and two for personal reasons. The remaining 24 participants completed all three sessions and were included in the analyses. EEG epochs containing stimulation artefacts were excluded according to the predefined analysis procedure.
Replication	Each participant completed three sessions.
Randomization	The order of the three sessions was randomized and counterbalanced across participants.
Blinding	Participants and the experimenter who interacted with participants were blinded to stimulation condition allocation.

Reporting for specific materials, systems and methods

We require information from authors about some types of materials, experimental systems and methods used in many studies. Here, indicate whether each material, system or method listed is relevant to your study. If you are not sure if a list item applies to your research, read the appropriate section before selecting a response.

Materials & experimental systems

n/a	Involved in the study
☒	☐ Antibodies
☒	☐ Eukaryotic cell lines
☒	☐ Palaeontology and archaeology
☒	☐ Animals and other organisms
☒	☐ Clinical data
☒	☐ Dual use research of concern
☒	☐ Plants

Methods

n/a	Involved in the study
☒	☐ ChIP-seq
☒	☐ Flow cytometry
☒	☐ MRI-based neuroimaging

Plants

Seed stocks	*Report on the source of all seed stocks or other plant material used. If applicable, state the seed stock centre and catalogue number. If plant specimens were collected from the field, describe the collection location, date and sampling procedures.*
Novel plant genotypes	*Describe the methods by which all novel plant genotypes were produced. This includes those generated by transgenic approaches, gene editing, chemical/radiation-based mutagenesis and hybridization. For transgenic lines, describe the transformation method, the number of independent lines analyzed and the generation upon which experiments were performed. For gene-edited lines, describe the editor used, the endogenous sequence targeted for editing, the targeting guide RNA sequence (if applicable) and how the editor was applied.*
Authentication	*Describe any authentication procedures for each seed stock used or novel genotype generated. Describe any experiments used to assess the effect of a mutation and, where applicable, how potential secondary effects (e.g. second site T-DNA insertions, mosiacism, off-target gene editing) were examined.*
