# Supplementary material for: Behaviourally driven closed-loop beta-tACS enhances beta activity and motor behaviour
Source: Nat Commun. 2026 Jul 21;17:8908. doi: 10.1038/s41467-026-75799-8 (PMC13503910; doi:10.1038/s41467-026-75799-8)
Supplement: Supplementary file 3 [file 41467_2026_75799_MOESM3_ESM.pdf]

nature portfolio

Peer Review File

Behaviourally driven closed-loop beta-tACS enhances beta activity and motor behaviour

Corresponding Author: Dr Min Wu

This file contains all reviewer reports in order by version, followed by all author rebuttals in order by version.

Version 0:

Reviewer comments:

Reviewer #1

(Remarks to the Author)
Wu et al. present an elegant and technically innovative study introducing a behaviourally triggered β-tACS paradigm designed to enhance post-movement beta synchronization (ERS) and improve motor learning. The study represents a significant advance in state-linked neuromodulation with clear translational potential.However, several interpretational and methodological issues require clarification and moderation.

1. Necessity of the closed-loop design
The manuscript emphasizes the novelty and importance of the closed-loop implementation. However, both active stimulation conditions ("early" and "late") were behaviourally triggered and both enhanced ERS and behavioural retention relative to sham, without differing significantly from one another.
Accordingly, while the results demonstrate that brief β-tACS bursts temporally linked to movement termination can improve retention, they do not establish that closed-loop timing is necessary for the observed effects. In the absence of an open-loop or continuous β-tACS control condition, the specific advantage of closed-loop delivery remains untested. Claims regarding the necessity or superiority of the closed-loop design should therefore be moderated.

2. Interpretation of causality and mechanism
The authors state that their results "establish a causal relationship between beta ERS and motor behaviour." This conclusion should be tempered.
Although stimulation increased post-movement beta power and improved behavioural retention, the EEG analyses were conducted on unstimulated trials to avoid contamination. Thus, the observed enhancement in ERS reflects a between-trial aftereffect rather than direct modulation of the ongoing ERS during stimulation. It remains unclear whether the stimulation directly altered the neural dynamics underlying the ERS itself or instead affected baseline activity or network states that influenced subsequent trials.
Consequently, the behavioural improvements could arise from mechanisms other than direct enhancement of ERS, such as increased cortical excitability, improved inter-trial consistency, or enhanced beta coherence during movement preparation. Conversely, higher ERS could simply reflect better performance, consistent with prior evidence linking stronger ERS to improved motor outcomes.
Taken together, the findings support a causal influence of β-tACS on behaviour, but not necessarily a causal relationship between endogenous ERS amplitude and motor retention. The discussion and abstract should acknowledge these alternative interpretations and soften the causal claim accordingly.

3. The design assumes that movement offset coincides reliably with beta ERS onset. However, the latency and duration of ERS can vary across subjects and trials, and may not align precisely with stimulation onset (particularly in the "late" condition).
The authors should provide evidence (e.g., group-averaged time-frequency plots) demonstrating that the stimulation window consistently overlapped with the natural ERS period, or else acknowledge this temporal uncertainty as a limitation.

4. In my opinion, it would be more appropriate, and stronger, to express the correlation between ERS change and behavioural retention relative to sham (i.e., stimulation-induced change) rather than using raw values. This would more directly test whether stimulation-driven modulation of ERS predicts the behavioural benefit.

5. Consider revising the title to explicitly convey that the paradigm employs a behaviourally driven, closed-loop β-tACS design.

6. Several clarifications would improve transparency and reproducibility:
• Make explicit in the results section that EEG analyses were restricted to non-stimulated trials.
• Specify the number and proportion of stimulated versus non-stimulated trials, and their distribution across force conditions.
• Report the number of epochs discarded due to stimulation or other artifacts.
• Include a description of the blinding questionnaire and clarify when it was administered.

Reviewer #2

(Remarks to the Author)
This is an exciting study that provides compelling evidence that tACS can modulate brain rhythms underlying motor performance. The study is well motivated, the experimental design is robust, and the results are convincing. I have a few questions and suggestions that may help further strengthen the manuscript:

Is it possible to estimate which cortical regions were specifically targeted by the tACS using e-field simulations?

Is the observed behavioural effect primarily driven by M1 (area 4), or could it instead reflect modulation of dorsal premotor regions?

Why did the authors not aim to stimulate premotor areas directly?

The sample size is small. How do the authors address interindividual variability and the possibility of responders vs. non-responders?

What is the rationale for stimulating at movement offset rather than movement onset? How might stimulation during movement execution have altered the effects?

Did age influence the stimulation effects in any way?

Reviewer #3

(Remarks to the Author)
This manuscript examines whether movement-related beta event-related synchronization (ERS) causally contributes to retention in a visuomotor adaptation task. The authors employ a double-blind, sham-controlled crossover design, delivering 20 Hz transcranial alternating current stimulation (tACS) over M1 under three conditions: (i) 2 seconds of beta-tACS at movement offset; (ii) beta-tACS commencing 2.5 seconds after movement offset; and (iii) a sham condition, with a brief 100 ms stimulation delivered 2.5 seconds after movement offset. They report that early beta-tACS enhances beta ERS in non stimulated trials without affecting beta event-related desynchronization (ERD), and that this enhancement is accompanied by improved retention. The study is clearly written, methodologically careful, and addresses a timely question in cognitive neuroscience.
Strengths
• The experimental question is novel and impactful, focusing on the timing-specific modulation of movement-related beta ERS and its causal contribution to a form of memory retention.
• The double-blind, sham-controlled crossover design and precise stimulation timing relative to movement offset are strong features.
• The results are presented clearly
Points to address to strengthen the manuscript
• Terminological precision: Please refer consistently to retention in a visuomotor adaptation task rather than to “skill” or “skill acquisition.” In the established literature, adaptation and skill memories denote distinct processes, and your paradigm aligns with adaptation and its retention.
• Clinical framing: The statement that this intervention could help “clinical populations characterized by impaired beta activity” is too broad and potentially misleading. Beta abnormalities are reported across diverse conditions, and their causal relevance to clinical symptoms remains uncertain. A more measured conclusion would be that the present approach offers a tool to test causal links (or their absence) between beta ERS and behavioral outcomes in clinical populations, rather than implying therapeutic efficacy at this stage.
• Stimulation parameters: I may have missed the intensity of tACS. Please specify the current amplitude (e.g., peak-to-peak or RMS in mA).
Minor suggestions
• Where claiming “causality,” consider moderating the language to “supports a causal contribution” and, if possible, include analyses that quantify null effects on ERD (e.g., equivalence tests or Bayes factors).
Conclusion This is an interesting and well-executed study that advances our understanding of the functional role of beta activity in memory retention in the context of an adaptation task. Addressing the points above—particularly the terminological precision, clinical framing, and stimulation parameters—will further strengthen the manuscript’s impact and clarity.

Version 1:

Reviewer comments:

Reviewer #1

(Remarks to the Author)
The authors have addressed the majority of my comments satisfactorily. The revisions have improved the clarity of the manuscript and strengthened the interpretation of the results.
One point that could be further clarified concerns the interpretation that stimulation modulates the relationship between post-movement beta ERS and retention (A_s). While this is suggested in the text, the absence of a significant relationship between stimulation-induced changes in ERS and A_s does not provide strong support for a change in brain–behavior coupling.
To more directly test this claim, I would recommend an ANCOVA-style analysis within a linear mixed-effects framework, including an interaction term between condition (stimulation vs sham) and beta ERS. This approach would formally assess whether the slope linking ERS to A_s differs between conditions, rather than relying on difference scores. I would therefore encourage the authors to formally assess this using an ANCOVA-style approach within a linear mixed-effects framework, including an interaction term between condition (stimulation vs sham) and beta ERS. This would allow testing whether the slope linking ERS to A_s differs between conditions, providing a more direct assessment of whether stimulation alters brain–behavior coupling.
For example:
As ~ beta_ERS * condition + (1 | subject)

If this interaction is not significant, it would suggest that stimulation does not reliably alter the ERS–retention relationship, helping to temper the current interpretation.

Reviewer #3

(Remarks to the Author)
My concerns have been addressed.

Open Access This Peer Review File is licensed under a Creative Commons Attribution 4.0 International License, which permits use, sharing, adaptation, distribution and reproduction in any medium or format, as long as you give appropriate credit to the original author(s) and the source, provide a link to the Creative Commons license, and indicate if changes were made.
In cases where reviewers are anonymous, credit should be given to 'Anonymous Referee' and the source.
The images or other third party material in this Peer Review File are included in the article’s Creative Commons license, unless indicated otherwise in a credit line to the material. If material is not included in the article’s Creative Commons license and your intended use is not permitted by statutory regulation or exceeds the permitted use, you will need to obtain permission directly from the copyright holder.
To view a copy of this license, visit https://creativecommons.org/licenses/by/4.0/

Response to Reviewers

We thank the reviewers for their helpful and constructive comments. We have carefully considered all of the issues raised and made extensive changes to the manuscript. We hope that they will agree that the manuscript has been considerably strengthened as a result.

We have responded to each of the points made individually below, including any relevant excerpts from the manuscript in each case. All changes to the manuscript have been highlighted in the revised document.

Reviewer 1

Wu et al. present an elegant and technically innovative study introducing a behaviourally triggered β-tACS paradigm designed to enhance post-movement beta synchronization (ERS) and improve motor learning. The study represents a significant advance in state-linked neuromodulation with clear translational potential. However, several interpretational and methodological issues require clarification and moderation.

We thank the Reviewer for their positive comments on the significance of the paper and pleased that they recognise the translational potential of this work. We are grateful for their thoughtful comments and have answered each of their points in detail below.

1. Necessity of the closed-loop design

The manuscript emphasizes the novelty and importance of the closed-loop implementation. However, both active stimulation conditions ("early" and "late") were behaviourally triggered and both enhanced ERS and behavioural retention relative to sham, without differing significantly from one another.

Accordingly, while the results demonstrate that brief β-tACS bursts temporally linked to movement termination can improve retention, they do not establish that closed-loop timing is necessary for the observed effects. In the absence of an open-loop or continuous β-tACS control condition, the specific advantage of closed-loop delivery remains untested. Claims regarding the necessity or superiority of the closed-loop design should therefore be moderated.

We thank the reviewer for this constructive point and agree that our data do not establish that closed-loop timing is necessary or superior to open-loop or continuous beta-tACS. While both active stimulation conditions were behaviourally triggered, the study was not designed to compare closed-loop stimulation against an open-loop or continuous control, and therefore we are happy to moderate our claims about the necessity or superiority of closed-loop delivery appropriately.

The late stimulation condition was included to provide a temporal comparison relative to movement offset; the 2.5 s delay was selected based on prior work indicating that post-movement beta ERS typically lasts approximately this duration (Wu et al., 2025). However, in the present visuomotor adaptation task, beta ERS was more prolonged than in previous studies, such that both early and late stimulation windows overlapped with the endogenous ERS. This likely explains why both conditions enhanced ERS and behavioural retention relative to sham, without differing significantly from one another.

Importantly, while these findings demonstrate that brief β-tACS bursts temporally linked to movement termination can enhance retention, they do not isolate the specific contribution of closed-loop delivery *per se*. Determining whether behavioural triggering confers advantages over open-loop or continuous stimulation will require future studies that directly compare these approaches while controlling for stimulation dose and timing.

In response to the reviewer's comment, we have added the following section to the discussion.

<u>Discussion (page 14):</u> "We observed effects for both early and late stimulation, which may reflect a prolonged beta ERS such that both stimulation windows overlapped with the ERS period. This does not rule out temporal specificity of beta-tACS; future studies should place stimulation clearly outside the ERS window (e.g., using a longer delay after movement offset) to more directly test temporal dependence. In addition, future work should directly compare closed-loop stimulation based on behaviour (as implemented here) with closed-loop stimulation based on neural activity, as well as with open-loop or continuous beta-tACS, to determine whether closed-loop delivery provides unique advantages beyond timing relative to endogenous beta activity."

2. Interpretation of causality and mechanism

The authors state that their results "establish a causal relationship between beta ERS and motor behaviour." This conclusion should be tempered. Although stimulation increased post-movement

beta power and improved behavioural retention, the EEG analyses were conducted on unstimulated trials to avoid contamination. Thus, the observed enhancement in ERS reflects a between-trial aftereffect rather than direct modulation of the ongoing ERS during stimulation. It remains unclear whether the stimulation directly altered the neural dynamics underlying the ERS itself or instead affected baseline activity or network states that influenced subsequent trials. Consequently, the behavioural improvements could arise from mechanisms other than direct enhancement of ERS, such as increased cortical excitability, improved inter-trial consistency, or enhanced beta coherence during movement preparation. Conversely, higher ERS could simply reflect better performance, consistent with prior evidence linking stronger ERS to improved motor outcomes.

Taken together, the findings support a causal influence of β-tACS on behaviour, but not necessarily a causal relationship between endogenous ERS amplitude and motor retention. The discussion and abstract should acknowledge these alternative interpretations and soften the causal claim accordingly.

We thank the reviewer for this insightful comment. We agree that our original phrasing did not acknowledge the potentially complex causal relationships between the increased beta ERS and behavioural improvements. As the Reviewer states, while our stimulation protocol increased post-movement beta power and improved behavioural retention, the EEG analyses were conducted on unstimulated trials to avoid contamination. As such, the observed enhancement in ERS reflects a between-trial aftereffect rather than direct modulation of ongoing ERS during stimulation.

To address the possibility that the stimulation merely altered baseline activity, we conducted a new analysis comparing beta power in the baseline window across conditions. As shown in the new Supplementary Fig. 2D, there was no significant difference in baseline beta power among stimulation conditions ($\chi^2(2) = 1.427$, $p = 0.49$). Furthermore, a Bayesian analysis yielded a BF_{01} of 35.645, providing strong evidence in favour of the null hypothesis (i.e., no difference in baseline state). This suggests that the observed behavioural effects are not simply driven by a non-specific shift in baseline cortical excitability or background beta levels.

It is theoretically possible, as the reviewer suggests, that some other mechanism, other than endogenous ERS, may contribute to the behavioural improvements. Although the manipulation between the stimulation and sham conditions was the application of beta tACS during the ERS,

and we have shown a clear difference in beta ERS between conditions, we cannot rule out changes in other neural signatures. If such changes exist, they are likely driven by the beta tACS as well. Given the precise targeting of the stimulation—applied at beta frequency during the post-movement beta ERS—any modulation of other neural signals and the observed changes in beta ERS likely share the same underlying mechanism. We have therefore tempered our claims in the Abstract and elaborated on this point in the Discussion, in line with the Reviewer's suggestion.

Results (page 6): "Crucially, a comparison of beta activity in the pre-movement baseline window revealed no significant differences across stimulation conditions ($\chi^2(2) = 1.427$, $p = 0.49$; $BF_{01} = 35.645$; Supplementary Fig. 2D)."

Abstract (page 2): "These findings support a functional role of beta ERS in motor behaviour and suggest that behaviourally-driven beta-tACS may provide a promising approach to further test the mechanistic links between beta ERS and behavioural outcomes in clinical populations."

Discussion (page 12): "However, since EEG analysis was restricted to unstimulated trials, the observed ERS enhancement reflects a between-trial aftereffect rather than direct online modulation of beta-tACS. This raises the question of whether the behavioural improvement is directly caused by the enhanced ERS or by other mechanisms induced by the beta stimulation. One possibility is that tACS altered the baseline neural state, such as general cortical excitability. However, our analysis of baseline beta power showed no differences between stimulation conditions, suggesting that the effect is not due to a global shift in baseline activity. Nevertheless, we cannot rule out that the stimulation modulates other unidentified neural signatures (e.g., brain connectivity) that concurrently improved behaviour and enhanced ERS, which was further supported by the null result that the changes of behaviour was not significantly predicted by the changes of beta ERS."

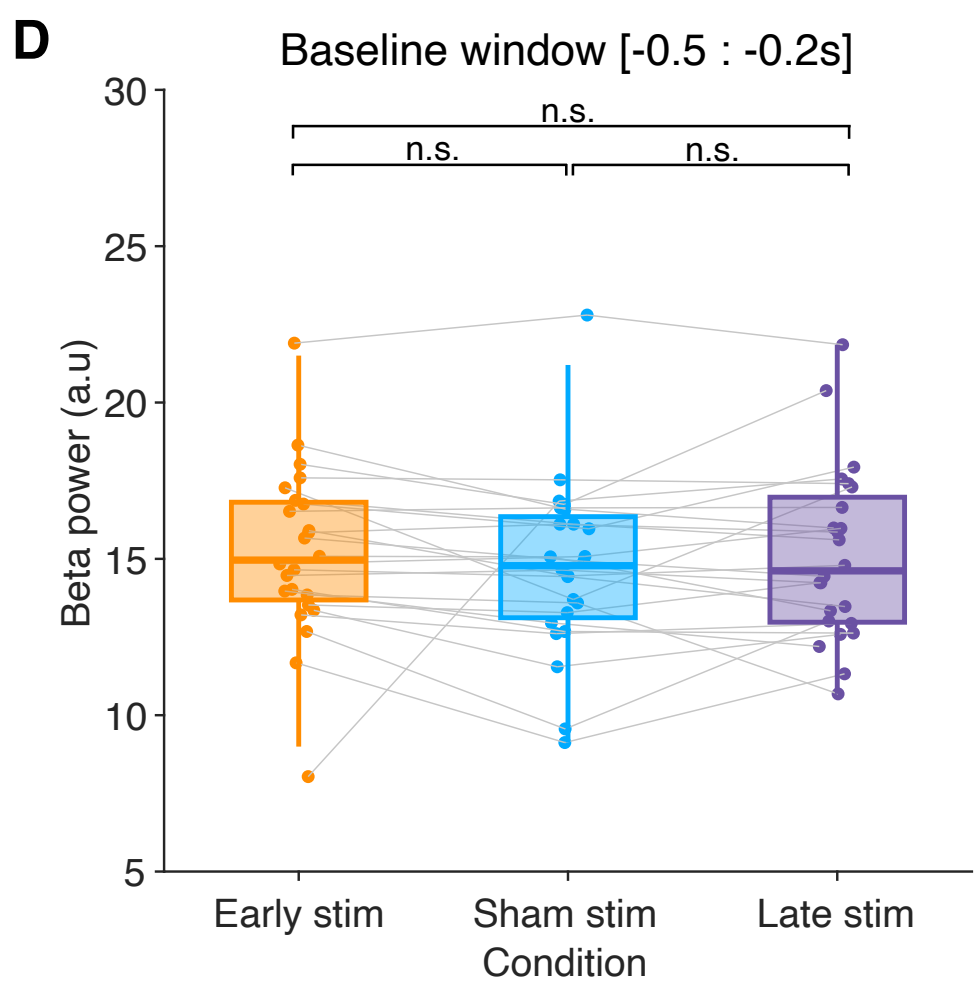

Supplementary Figure 2D. Mean beta power extracted from pre-movement baseline window over the contralateral sensorimotor area.

3. The design assumes that movement offset coincides reliably with beta ERS onset. However, the latency and duration of ERS can vary across subjects and trials, and may not align precisely with stimulation onset (particularly in the "late" condition).

The authors should provide evidence (e.g., group-averaged time-frequency plots) demonstrating that the stimulation window consistently overlapped with the natural ERS period, or else acknowledge this temporal uncertainty as a limitation.

We thank the reviewer for this constructive comment. We agree that the latency and duration of the post-movement beta ERS can vary across participants and trials, and therefore behavioural triggering at movement offset cannot guarantee perfect alignment with ERS onset on every trial.

To address this concern, we have provided two complementary pieces of evidence demonstrating that, despite trial-to-trial variability, our stimulation overlapped with the natural post-movement beta ERS at both the group and individual levels. First**,** we present group-averaged time–frequency responses aligned to the behavioural event (target reached [t_0]) used for triggering (Fig. 2). The post-movement beta ERS is clearly visible in the expected time–frequency window over contralateral sensorimotor areas (dashed rectangles). Importantly, the stimulation windows are shown on the same plots (sinusoidal waveforms; Fig. 2A early, Fig. 2B

late), showing that both the early and late stimulation epochs overlap with the broader post-movement beta ERS period at the group level.

Second, to account for inter-individual variability, we have now added an individual-level visualization (Supplementary Fig. 6) showing the estimated ERS timing for each participant and its overlap with the early and late stimulation windows. This plot shows that although ERS timing varies across individuals, the stimulation windows overlap with the participant-specific ERS period in the substantial majority of participants in both conditions, while also making explicit that overlap is not perfect in all individuals.

Finally, we have updated the *Discussion* to acknowledge this temporal uncertainty explicitly (page 13): "However, while behavioural triggering increases the likelihood of stimulating during the intended post-movement beta state, ERS onset and duration can vary across participants and trials, such that alignment with the endogenous beta state is approximate rather than exact on every trial. This variability further motivates future approaches that combine behavioural and neural signals to guide closed-loop stimulation."

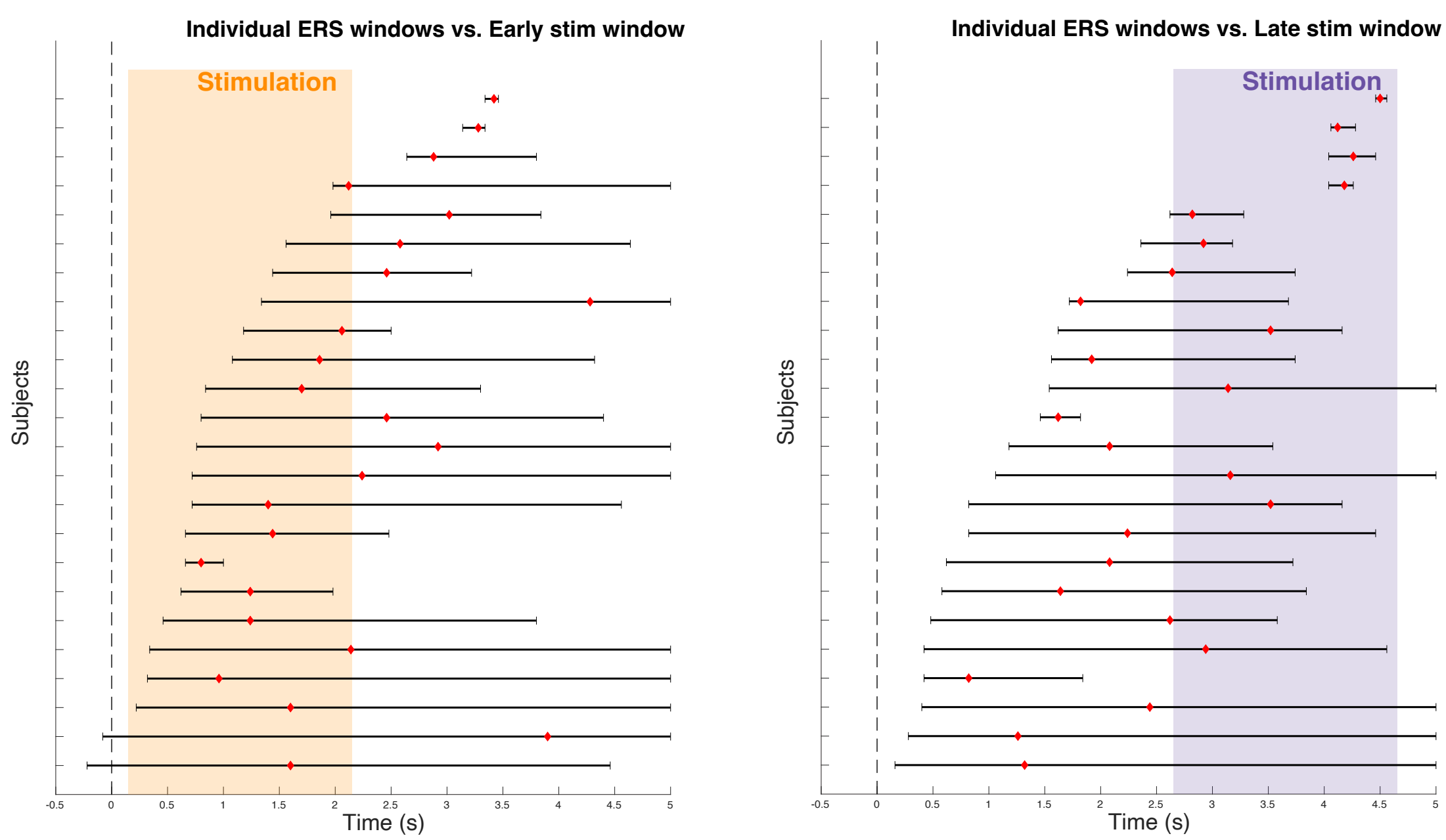

Supplementary Figure 6. Individual post-movement beta ERS windows and stimulation windows. The black horizontal line indicates the ERS window (onset to offset), and the red dots denotes the latency of the ERS peak.

4. In my opinion, it would be more appropriate, and stronger, to express the correlation between ERS change and behavioural retention relative to sham (i.e., stimulation-induced change) rather than using raw values. This would more directly test whether stimulation-driven modulation of ERS predicts the behavioural benefit.

We thank the reviewer for this suggestion. While we agree that this would be an interesting approach, this approach assumes a high degree of reproducibility in the behaviour and EEG metrics from day-to-day. While we have made every effort to minimise variability and improve reproducibility, between-day comparisons are inherently noisier than those acquired on the same day. Further, this would assume a linear relationship between improvement in ERS and behavioural benefits, which may rather suffer from ceiling and floor effects in both metrics.

However, to test whether the stimulation-related behavioural benefit was specifically explained by stimulation-related modulation in beta ERS, we re-analysed the data using difference scores (stimulation – sham) for both ERS and retention. The linear mixed-effects model revealed that changes of beta ERS did not significantly predict the changes of As ($p > 0.05$). This result indicates that although higher post-movement beta ERS is associated with greater retention overall, the magnitude of stimulation-driven ERS increase does not linearly account for the magnitude of behavioural improvement across participants and sessions.

This dissociation may reflect the possibility that stimulation affects retention through multiple mechanisms not captured by broad-window ERS amplitude alone, which supported the point 2 raised by the Reviewer. We have added this clarification to Results and the Discussion to explicitly acknowledge the null correlation.

Results (page 8): "Nevertheless, no significant correlation was observed between stimulation-induced changes in beta ERS and A_s ($\beta = 0.0005$, $p = 0.868$), indicating that inter-individual variation in ERS modulation did not linearly predict the magnitude of behavioural benefit in this sample."

Discussion (page 12): "Nevertheless, we cannot rule out that the stimulation modulates other unidentified neural signatures (e.g., brain connectivity) that concurrently improved behaviour and enhanced ERS, which was further supported by the null result that the changes of behaviour was not significantly predicted by the changes of beta ERS."

5. Consider revising the title to explicitly convey that the paradigm employs a behaviourally driven, closed-loop β-tACS design.

Thank you. We have revised the title as suggested to explicitly highlight the behaviourally driven, closed-loop β-tACS design to the following: "Behaviourally driven closed-loop beta-tACS enhances beta activity and motor behaviour".

6. Several clarifications would improve transparency and reproducibility:

• Make explicit in the results section that EEG analyses were restricted to non-stimulated trials.

We have now made this explicit in the *Results* (page 5): "EEG was recorded continuously during the visuomotor adaptation task and was analysed from those trials where tACS was not applied; all subsequent EEG analyses are based on non-stimulated epochs."

• Specify the number and proportion of stimulated versus non-stimulated trials, and their distribution across force conditions.

Stimulation and non-stimulation trials followed a deterministic repeating pattern of four stimulated trials followed by two non-stimulated trials (1–1–1–1–0–0), so that two out of every six trials (33.3%) were intentionally left unstimulated to provide artifact-free trials for EEG analysis. This pattern was applied continuously throughout the session. Because the start of the sequence was randomized across participants, the exact number of non-stimulated trials varied slightly based on the starting trial, ranging between 130-132.

Force-field magnitudes followed the sequence: *0, 3f, 6f, 9f, 6f, 3f, 0*, which was repeated four times, with 14 trials per force field, resulting in 56 trials at each force level. Considering the continuous 1–1–1–1–0–0 pattern, the number of non-stimulated trials per force level was 18-20.

We have provided these numbers in *Methods* (page 18); as "tACS was applied in four out of every six trials, with the remaining two trials left stimulation-free and thus artifact-free for EEG analysis. Therefore for each force level 18-20 of the 56 trials were stimulation-free, resulting in a total of 130–132 stimulation-free trials per session (Supplementary Fig. 4)."

We additionally provide a figure showing the distribution of stimulated versus non-stimulated trials across force fields in Supplementary Fig. 4.

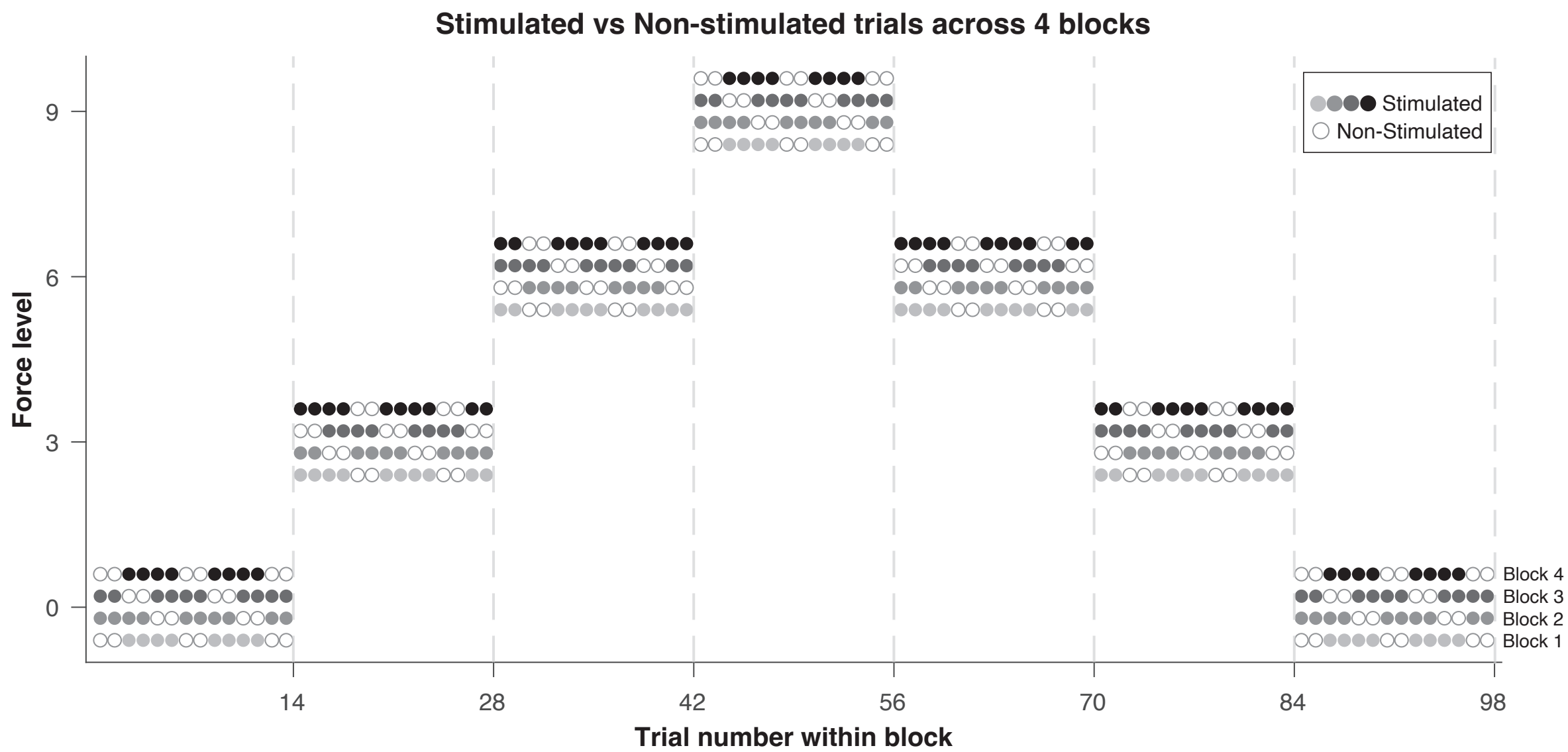

Supplementary Figure 4. Distribution of stimulated and non-stimulated trials across force fields for a representative session. Each block consists of 98 trials (14 trials × 7 force levels: 0, 3, 6, 9, 6, 3, 0). The four repetitions of the block are stacked.

• Report the number of epochs discarded due to stimulation or other artifacts.

A total of 114 ± 19 (mean ± SD) were included in the EEG analysis. We have now included this into the *Methods* (page 21).

• Include a description of the blinding questionnaire and clarify when it was administered.

We thank the reviewer for this suggestion. To assess blinding efficacy, immediately after each session, both the participant and the interacting experimenter completed a brief questionnaire indicating their belief about whether the session involved active or sham stimulation. Responses were recorded on a 5-point scale: 1 ("I strongly believe the treatment was active stimulation"), 2 ("I somewhat believe the treatment was active stimulation"), 3 ("I somewhat believe the treatment was sham"), 4 ("I strongly believe the treatment was placebo"), and 5 ("I don't know"). Participants were informed that the study included both active and sham stimulation but were not provided with details regarding expected sensations. Experimenters rated their beliefs based

on observations of the participant. This procedure ensured that blinding could be evaluated for both participants and experimenters.

We have added the following to the Methods (page 15): "To assess blinding efficacy, immediately after each session, both the participant and the interacting experimenter completed a brief blinding questionnaire indicating their belief about whether the session involved active or sham stimulation using a 5-point scale (1 = strongly active, 2 = somewhat active, 3 = somewhat sham, 4 = strongly sham, 5 = don't know)."

Reviewer 2

This is an exciting study that provides compelling evidence that tACS can modulate brain rhythms underlying motor performance. The study is well motivated, the experimental design is robust, and the results are convincing. I have a few questions and suggestions that may help further strengthen the manuscript.

We thank the Reviewer for their very positive comments and for their constructive suggestions on the manuscript.

1. Is it possible to estimate which cortical regions were specifically targeted by the tACS using e-field simulations?

We thank the Reviewer for this valuable suggestion. To estimate the cortical regions targeted by tACS, we performed electric field (e-field) simulations using SimNIBS (v 4.5.0). We used the SimNIBS build-in head model Ernie to estimate the distribution of the induced electric field. The electrodes were modelled as circular pads (radius 1 cm, thickness 1 mm) with a 5-mm gel layer. Tissue conductivities were set to SimNIBS default values (scalp: 0.465 S/m, skull: 0.01 S/m, cerebrospinal fluid: 1.654 S/m, gray matter: 0.275 S/m, white matter: 0.126 S/m). The results indicate that the stimulation primarily engaged the left sensorimotor cortex, consistent with the electrode montage used in the study (see Supplementary Fig. 5 below). This figure has now been included in the Supplementary Information of the revised manuscript. These simulations confirm that the applied tACS likely modulated neural activity in the intended motor-related cortical region.

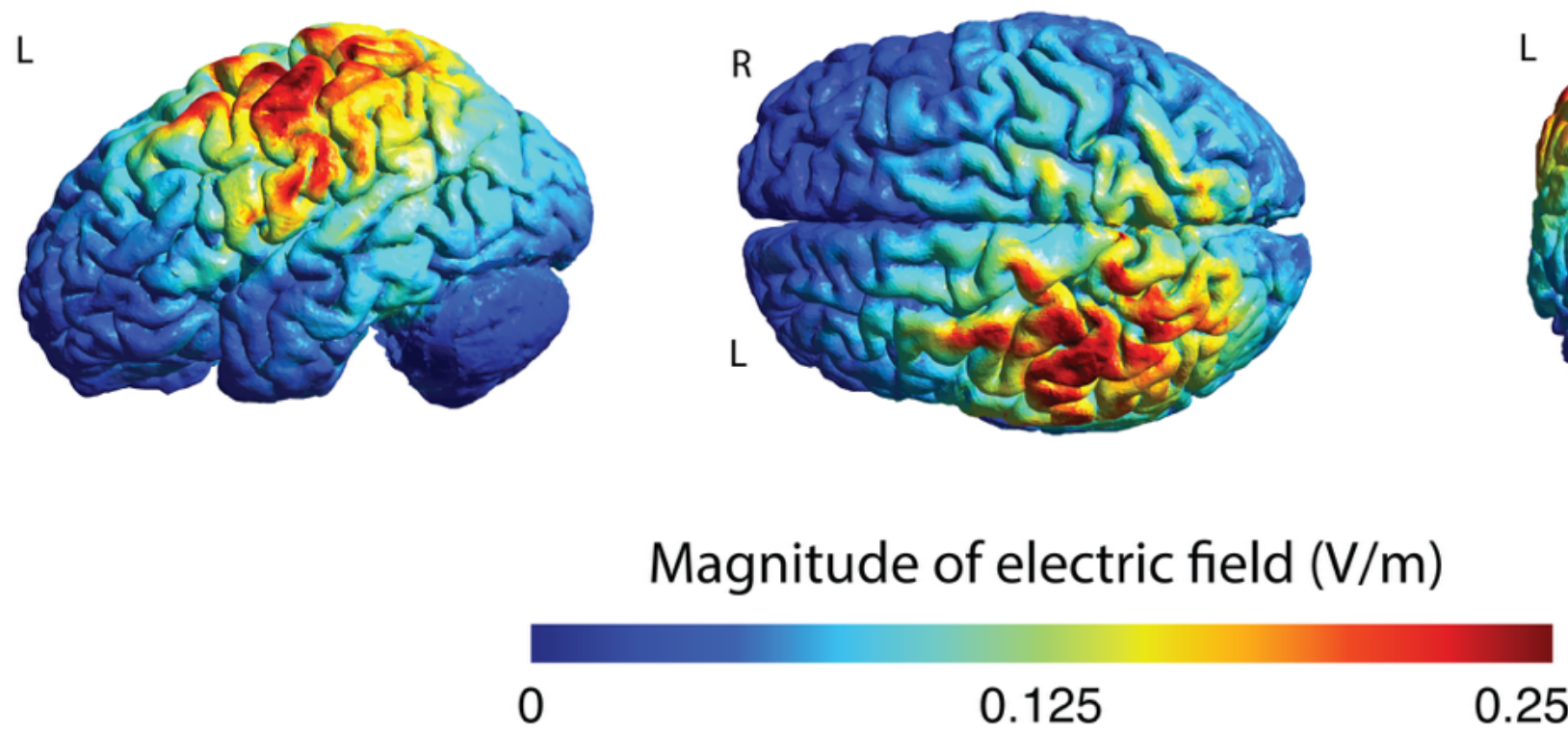

Supplementary Figure 5. Electric field simulation of C3-Pz tACS.

Methods (page 17): "To estimate the spatial distribution and magnitude of the induced electric field in the brain, we performed simulations using the SimNIBS software toolbox (version 4.5.0) with the build-in head model Ernie [51]".

2. Is the observed behavioural effect primarily driven by M1 (area 4), or could it instead reflect modulation of dorsal premotor regions?

This is a very interesting point, and something that we have now explored. The simulation shown in the new Supplementary Figure 5 above, demonstrates that the stimulation was indeed centred on the hand knob of M1. To quantify the intensity of the stimulation in M1 and pMd, we then performed E-field simulations using SimNIBS (v4.5.0) and extracted regional magnitudes using the HCP-MMP1 atlas. The analysis confirms that our montage, designed to target the motor cortex, successfully delivered the maximal electric field intensity to the Primary Motor Cortex (M1, Area 4), with the peak electric field magnitude in Area 4 of 0.28 V/m exceeding the peak intensity in the dorsal premotor cortex (Area 6d: 0.25 V/m). Accordingly, we interpret the observed behavioural effects as primarily reflecting modulation of M1 activity, although we cannot rule out contributions from dorsal premotor areas. We added the following sentence to the Methods section of the manuscript to address this point (Page 18):

"Electric field simulations confirmed that the peak field intensity occurred in left sensorimotor cortex (Supplementary Fig. 5), while the stimulation also engaged the adjacent dorsal premotor cortex."

3. Why did the authors not aim to stimulate premotor areas directly?

We thank the reviewer for this insightful point. Premotor areas are indeed plausible stimulation targets and have been associated with motor learning and retention (e.g., (Hadipour-Niktarash et al., 2007; Kantak et al., 2012; Sugiyama et al., 2024)), making them an interesting target. However, our decision not to target premotor cortex directly was due to the following considerations:

1. Our *a priori* mechanistic target was the post-movement beta ERS, which is most robustly expressed over the contralateral sensorimotor and motor cortex and can be reliably measured with scalp EEG in this region (Espenhahn et al., 2017; Parkes et al., 2006; Pfurtscheller & Lopes da Silva, 1999).

2. The primary motor cortex (M1) has been consistently linked to motor retention (Hadipour-Niktarash et al., 2007; Reis et al., 2009).

We agree that a direct comparison between M1-targeted and premotor-targeted stimulation represents an important next step to explore the spatial specificity of these effects. We have added the following to the *Discussion* (pages 13-14): "Furthermore, although the present study targeted M1, other regions involved in motor control and learning, such as premotor cortex, may also contribute to performance. Future work should compare M1-targeted stimulation with other motor cortical regions, such as premotor cortex, to determine the regional specificity of beta activity and its contribution to motor retention."

4. The sample size is small. How do the authors address interindividual variability and the possibility of responders vs. non-responders?

We agree that the sample size is relatively small (N = 24, within-subject, cross-over across three sessions, totalling n = 72 sessions) and that inter-individual variability is an important consideration in tACS studies. We designed our experiment in the following ways to address this inherent potential issue:

1. We performed a repeated-measures crossover design: Each participant completed all three conditions (Early Stim, Late Stim, Sham), allowing every participant to serve as their own control. This approach increases statistical power and accounts for baseline inter-individual differences.

2. We used Linear Mixed Models (LMMs): Random intercepts for each subject (1 | Subject) were included to account for between-subject variability.

3. Visualization of individual responses: Subject-level plots (e.g., Figure 2) show that the direction of the stimulation effect was consistent across the majority of participants, arguing against effects driven by a small subset.

However, it is important to ensure that the effects we see are not driven by a single participant. We have therefore now additionally conducted a leave-one-out cross-validation (LOOCV), refitting the mixed-effects model 24 times, each time excluding one participant. The stimulation effect remained significant across all iterations, demonstrating robustness to single-subject influence.

Results (page 8): "We have shown that tACS leads to an increase in beta ERS amplitude, and to a specific improvement in retention. This stimulation effect was further verified by a leave-one-participant-out sensitivity analysis (*p ranges: ERS at early window: 0.003–0.025; ERS at late window: 0.001–0.009; As: 0.002–0.012*), by refitting the mixed-effects model 24 times, each time excluding one participant."

5. What is the rationale for stimulating at movement offset rather than movement onset? How might stimulation during movement execution have altered the effects?

We chose movement offset as the trigger because our primary mechanistic target was the post-movement beta ERS, rather than beta ERD during movement execution. Post-movement beta ERS has been linked to functionally meaningful processes such as updating of internal models and error-related processing during motor learning and adaptation (Tan et al., 2014; Wu et al., 2025). Our closed-loop design was therefore intended to boost endogenous post-movement beta ERS by delivering beta-tACS after movement.

A second motivation is translational relevance: post-movement beta ERS in motor cortex is often altered in populations with motor impairment, including stroke, and has been associated with future motor recovery (Laaksonen et al., 2012; Zich et al., 2025). Targeting this physiologically meaningful marker increases the potential clinical relevance of our approach.

Stimulation during movement execution would engage a different physiological process. During movement, beta power is suppressed, and conventional beta-tACS is not optimized to further suppress beta activity (i.e., increase ERD [less negative]). Achieving reliable suppression would

likely require anti-phase stimulation with accurate real-time phase estimation and robust artifact removal, which is technically challenging requiring the synchronization of neural signals, behaviour performance and brain stimulation.

If beta-tACS were applied during movement execution, we would expect effects on movement speed rather than movement accuracy (lateral deviation) observed in this study. Increasing beta synchrony during ongoing movement has been associated with reduced movement vigor and maintenance of the current motor state, and causal evidence shows that 20 Hz stimulation over motor cortex can slow voluntary movements (Joundi et al., 2012; Pogosyan et al., 2009). Thus, delivering beta-tACS during movement could plausibly attenuate ERD (make it less negative) and reduce movement velocity.

6. Did age influence the stimulation effects in any way?

We thank the reviewer for raising this important point. Although our participants were young adults (18–35 years) with limited age variability, we tested whether age influenced stimulation effects by re-running the linear mixed-effects models for all neural and behavioural outcomes, including both the main effect of age and the age × stimulation condition interaction. Across all outcomes, there were no significant main effects of age and no age × stimulation condition interactions (all $p > 0.05$). Furthermore, likelihood ratio tests further confirmed that adding age did not significantly improve model fit compared to the original models. These analyses indicate that the effects of closed-loop stimulation are robust and independent of age within the range tested.

However, given the limited age range of our sample, these effects require further validation in older populations (Opie et al., 2017). We have added a brief discussion:

Discussion (page 13): "Importantly, although age did not significantly influence any behavioural or neural outcomes in our study (all $p > 0.05$), our sample was restricted to young adults. Therefore, generalization to older adults and potential age-related modulation of the effects require further study."

Table R1. Age-covariate analyses

Outcome ~ Stim condition × Age + (1 | Subject)				
Outcome	Stim condition	Age	Age × Stim condition	Likelihood ratio (model with vs. without Age)
ERS at early window	p = 0.008	p = 0.211	p = 0.507	p = 0.570
ERS at late window	p = 0.003	p = 0.128	p = 0.937	p = 0.358
Lateral deviation	p = 0.019	p = 0.466	p = 0.933	p = 0.889
As	p = 0.003	p = 0.935	p = 0.977	p = 0.979

Reviewer 3

This manuscript examines whether movement-related beta event-related synchronization (ERS) causally contributes to retention in a visuomotor adaptation task. The authors employ a double-blind, sham-controlled crossover design, delivering 20 Hz transcranial alternating current stimulation (tACS) over M1 under three conditions: (i) 2 seconds of beta-tACS at movement offset; (ii) beta-tACS commencing 2.5 seconds after movement offset; and (iii) a sham condition, with a brief 100 ms stimulation delivered 2.5 seconds after movement offset. They report that early beta-tACS enhances beta ERS in non stimulated trials without affecting beta event-related desynchronization (ERD), and that this enhancement is accompanied by improved retention. The study is clearly written, methodologically careful, and addresses a timely question in cognitive neuroscience.

Strengths

• The experimental question is novel and impactful, focusing on the timing-specific modulation of movement-related beta ERS and its causal contribution to a form of memory retention.

• The double-blind, sham-controlled crossover design and precise stimulation timing relative to movement offset are strong features.

• The results are presented clearly

We thank the Reviewer for their clear summary of the paper and for their helpful suggestions to strengthen the manuscript.

Points to address to strengthen the manuscript

1. Terminological precision: Please refer consistently to retention in a visuomotor adaptation task rather than to "skill" or "skill acquisition." In the established literature, adaptation and skill memories denote distinct processes, and your paradigm aligns with adaptation and its retention.

Thank you for this point. We fully agree that "retention" in a visuomotor adaptation task should be used consistently, rather than "skill" or "skill acquisition," because adaptation and skill learning are distinct processes. We have updated the manuscript throughout to ensure terminological consistency and revised the discussion paragraph accordingly, removing references to skill learning.

For example, in Introduction (page 4): "We hypothesized that behaviourally-triggered, closed-loop beta tACS delivered at movement offset (i.e., the early stimulation condition) would enhance beta ERS in the sensorimotor cortex and facilitate ~~the acquisition of a motor skill~~ motor performance in a visuomotor adaptation task."

Discussion (pages 11-12): "In our study, applying beta tACS immediately after the movement increased post-movement beta ERS, and this increase was associated with larger retention factor in the slow process. Our results align with recent findings demonstrating that 20 Hz tACS over the motor cortex after training enhances retention of performance measured after 24-hours and 7-days compared to sham stimulation [39]. Similarly, previous studies have shown that beta-tACS increases corticomuscular coherence, which positively correlates with improved motor retention [18]. The relationship between beta ERS and retention was also observed in observational studies. For example, healthy individuals who exhibit larger increases in beta ERS during practice typically show stronger retention of motor performance. In contrast, patients with Parkinson's disease show smaller beta ERS and impaired motor retention [40]."

2. Clinical framing: The statement that this intervention could help "clinical populations characterized by impaired beta activity" is too broad and potentially misleading. Beta abnormalities are reported across diverse conditions, and their causal relevance to clinical symptoms remains uncertain. A more measured conclusion would be that the present approach

offers a tool to test causal links (or their absence) between beta ERS and behavioral outcomes in clinical populations, rather than implying therapeutic efficacy at this stage.

Thank you, we have now revised the Abstract to emphasise the mechanistic investigation, as suggested:

"These findings support a functional role of beta ERS in motor behaviour and suggest that behaviourally-driven beta-tACS may provide a promising approach to further test the mechanistic links between beta ERS and behavioural outcomes in clinical populations."

3. Stimulation parameters: I may have missed the intensity of tACS. Please specify the current amplitude (e.g., peak-to-peak or RMS in mA).

Stimulation was applied with the current amplitude of 2mA peak-to-peak (i.e. between ±1mA; page 17). In the revised manuscript, we have also included the corresponding electric field simulations (Supplementary Figure 5) to provide additional information about the spatial distribution and intensity of the applied tACS.

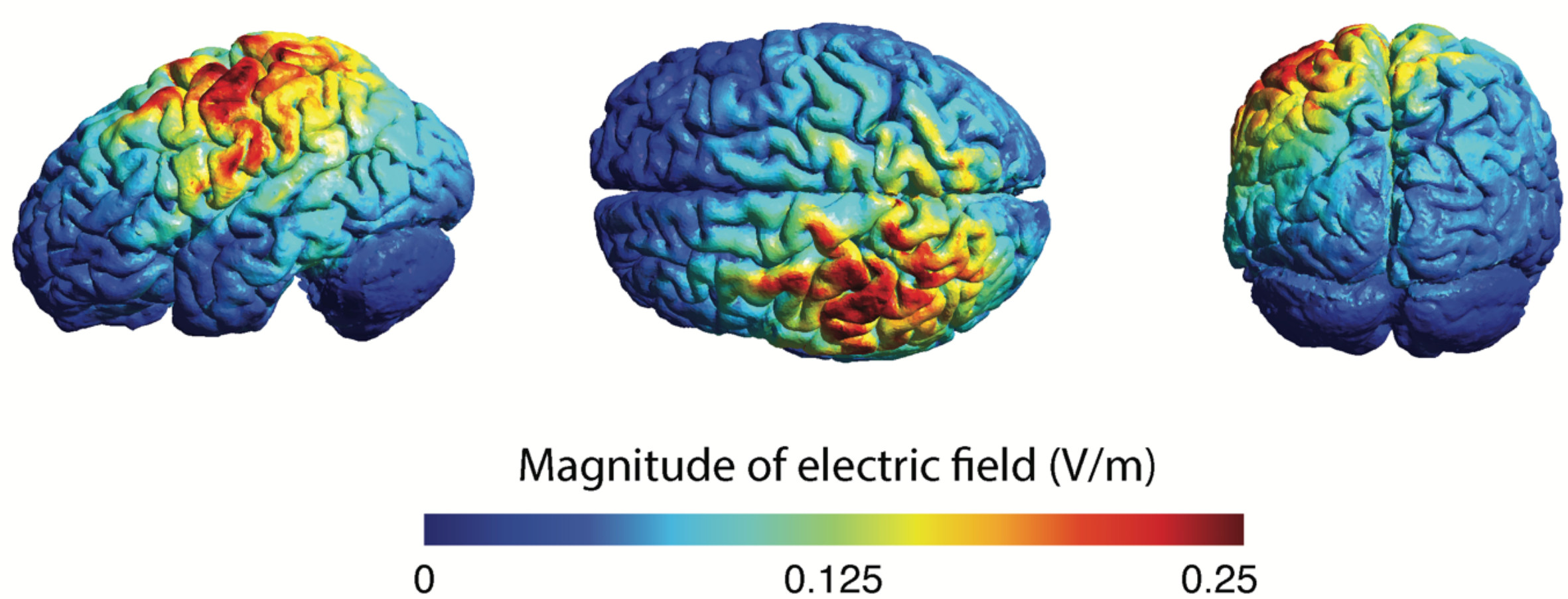

Supplementary Figure 5. Electric field simulation of C3-Pz tACS.

4. Where claiming "causality," consider moderating the language to "supports a causal contribution" and, if possible, include analyses that quantify null effects on ERD (e.g., equivalence tests or Bayes factors).

We thank the reviewer for this important suggestion. We have moderated the language from "establishes causality" to "supports a causal contribution". Furthermore, to explicitly quantify

evidence for null effects on beta ERD, we calculated the Bayes Factor (BF_{01}) comparing the linear mixed model with and without the stimulation condition. This analysis yielded BF_{01} = 20.16, indicating strong evidence in favor of the null hypothesis. This confirms that the movement-related beta ERD was unaffected by our closed-loop intervention.

We have added this into the *Results* (page 6): "To determine whether these stimulation-related changes were specific to the beta ERS, we investigated the amplitude of the beta ERD in a similar way….when the mean amplitude of the beta power within the movement period (0–1.5 s relative to *movement onset*) was calculated and compared across stimulation conditions, no significant differences in beta ERD were observed ($\chi^2(2) = 2.615$, $p = 0.271$; Extended Data Fig. 2). This null effect was further supported by a Bayes Factor (BF_{01} = 20.160), indicating strong evidence for the absence of stimulation effects on movement-related beta ERD."

Conclusion: This is an interesting and well-executed study that advances our understanding of the functional role of beta activity in memory retention in the context of an adaptation task. Addressing the points above—particularly the terminological precision, clinical framing, and stimulation parameters—will further strengthen the manuscript's impact and clarity.

We thank the reviewer for these positive comments and constructive suggestions. We believe these revisions have highly improved the manuscript.

References:

Espenhahn, S., de Berker, A. O., van Wijk, B. C. M., Rossiter, H. E., & Ward, N. S. (2017). Movement-related beta oscillations show high intra-individual reliability. *NeuroImage*, *147*, 175–185. https://doi.org/10.1016/j.neuroimage.2016.12.025

Hadipour-Niktarash, A., Lee, C. K., Desmond, J. E., & Shadmehr, R. (2007). Impairment of Retention But Not Acquisition of a Visuomotor Skill Through Time-Dependent Disruption of Primary Motor Cortex. *The Journal of Neuroscience*, *27*(49), 13413–13419. https://doi.org/10.1523/JNEUROSCI.2570-07.2007

Joundi, R. A., Jenkinson, N., Brittain, J.-S., Aziz, T. Z., & Brown, P. (2012). Driving Oscillatory Activity in the Human Cortex Enhances Motor Performance. *Current Biology*, *22*(5), 403–407. https://doi.org/10.1016/j.cub.2012.01.024

Kantak, S. S., Mummidisetty, C. K., & Stinear, J. W. (2012). Primary motor and premotor cortex in implicit sequence learning – evidence for competition between implicit and explicit human motor memory systems. *European Journal of Neuroscience*, *36*(5), 2710–2715. https://doi.org/10.1111/j.1460-9568.2012.08175.x

Laaksonen, K., Kirveskari, E., Mäkelä, J. P., Kaste, M., Mustanoja, S., Nummenmaa, L., Tatlisumak, T., & Forss, N. (2012). Effect of afferent input on motor cortex excitability during stroke recovery. *Clinical Neurophysiology*, *123*(12), 2429–2436. https://doi.org/10.1016/j.clinph.2012.05.017

Opie, G. M., Vosnakis, E., Ridding, M. C., Ziemann, U., & Semmler, J. G. (2017). Priming theta burst stimulation enhances motor cortex plasticity in young but not old adults. *Brain Stimulation*, *10*(2), 298–304. https://doi.org/10.1016/j.brs.2017.01.003

Parkes, L. M., Bastiaansen, M. C. M., & Norris, D. G. (2006). Combining EEG and fMRI to investigate the post-movement beta rebound. *NeuroImage*, *29*(3), 685–696. https://doi.org/10.1016/j.neuroimage.2005.08.018

Pfurtscheller, G., & Lopes da Silva, F. H. (1999). Event-related EEG/MEG synchronization and desynchronization: Basic principles. *Clinical Neurophysiology*, *110*(11), 1842–1857. https://doi.org/10.1016/S1388-2457(99)00141-8

Pogosyan, A., Gaynor, L. D., Eusebio, A., & Brown, P. (2009). Boosting Cortical Activity at Beta-Band Frequencies Slows Movement in Humans. *Current Biology*, *19*(19), 1637–1641. https://doi.org/10.1016/j.cub.2009.07.074

Reis, J., Schambra, H. M., Cohen, L. G., Buch, E. R., Fritsch, B., Zarahn, E., Celnik, P. A., & Krakauer, J. W. (2009). Noninvasive cortical stimulation enhances motor skill acquisition over multiple days through an effect on consolidation. *Proceedings of the National Academy of Sciences*, *106*(5), 1590–1595. https://doi.org/10.1073/pnas.0805413106

Sugiyama, T., Uehara, S., & Izawa, J. (2024). Meta-learning of human motor adaptation via the dorsal premotor cortex. *Proceedings of the National Academy of Sciences*, *121*(44), e2417543121. https://doi.org/10.1073/pnas.2417543121

Tan, H., Jenkinson, N., & Brown, P. (2014). Dynamic Neural Correlates of Motor Error Monitoring and Adaptation during Trial-to-Trial Learning. *The Journal of Neuroscience*, *34*(16), 5678–5688. https://doi.org/10.1523/JNEUROSCI.4739-13.2014

Wu, M., Schoenfeld, M. J., Lindersson, C., Braeutigam, S., Zich, C., & Stagg, C. J. (2025). Differential Beta and Gamma Activity Modulation during Unimanual and Bimanual

Motor Learning. *Journal of Neuroscience*, *45*(22).
https://doi.org/10.1523/JNEUROSCI.2187-24.2025

Zich, C., Ward, N. S., Forss, N., Bestmann, S., Quinn, A. J., Karhunen, E., & Laaksonen, K. (2025). Post-stroke changes in brain structure and function can both influence acute upper limb function and subsequent recovery. *NeuroImage: Clinical*, *45*, 103754.
https://doi.org/10.1016/j.nicl.2025.103754

Reviewer #1:

The authors have addressed the majority of my comments satisfactorily. The revisions have improved the clarity of the manuscript and strengthened the interpretation of the results.
One point that could be further clarified concerns the interpretation that stimulation modulates the relationship between post-movement beta ERS and retention (A_s). While this is suggested in the text, the absence of a significant relationship between stimulation-induced changes in ERS and A_s does not provide strong support for a change in brain–behavior coupling.
To more directly test this claim, I would recommend an ANCOVA-style analysis within a linear mixed-effects framework, including an interaction term between condition (stimulation vs sham) and beta ERS. This approach would formally assess whether the slope linking ERS to A_s differs between conditions, rather than relying on difference scores. I would therefore encourage the authors to formally assess this using an ANCOVA-style approach within a linear mixed-effects framework, including an interaction term between condition (stimulation vs sham) and beta ERS. This would allow testing whether the slope linking ERS to A_s differs between conditions, providing a more direct assessment of whether stimulation alters brain–behavior coupling.
For example:
*As ~ beta_ERS * condition + (1 | subject)*

If this interaction is not significant, it would suggest that stimulation does not reliably alter the ERS–retention relationship, helping to temper the current interpretation.

We thank the reviewer for this helpful and constructive suggestion. We performed the recommended ANCOVA-style linear mixed-effects analysis to directly test whether the slope linking beta ERS to A_s differed across stimulation conditions. This analysis did not reveal a significant "*beta ERS* × *condition*" interaction ($\chi^2(2) = 4.388$, $p = 0.112$), indicating that the slope relating beta ERS to A_s did not differ reliably between stimulation and sham conditions.

This result is consistent with the difference-score analysis added in the first revision, in which stimulation-induced changes in beta ERS did not significantly predict stimulation-induced changes in A_s. Together, these analyses indicate that although stimulation increased beta ERS and improved retention, the current data do not provide strong evidence that stimulation reliably altered the ERS–retention relationship itself. We would suggest, however, that this does not necessarily argue against our underlying hypothesis that greater beta ERS leads to greater

behavioural retention. If beta tACS increases the beta ERS in a behaviourally-relevant way then we would not expect to see a difference in the slope of the relationship between beta ERS and A_S between conditions as we have not changed the nature of this relationship, but rather the stimulation has increased both beta ERS and A_S.

In light of the Reviewer's comments we have revised the *Results* and *Discussion* to temper this coupling interpretation accordingly.

<u>Results (page 8):</u> "However, the beta ERS × stimulation condition interaction was not significant ($\chi^2(2) = 4.388$, $p = 0.112$), indicating that the slope relating beta ERS to A_s did not differ reliably between conditions."

<u>Discussion (pages 12-13):</u> "Nevertheless, we cannot rule out that the stimulation modulates other unidentified neural signatures (e.g., brain connectivity) that concurrently improved behaviour and enhanced ERS. This interpretation is further supported by the finding that changes in beta ERS did not significantly predict changes in A_s. In addition, the mixed-effects analysis did not provide evidence that stimulation reliably altered the relationship between beta ERS and A_s."
